# Intravascular Imaging-Guided Drug-Eluting Stenting for Complex Coronary Lesions: A Meta-Analysis of Randomized Controlled Trials

**DOI:** 10.1016/j.jacadv.2026.103340

**Published:** 2026-10-15

**Authors:** Mohammed A. Elbahloul, Bahaa El Deen Wehbeh, Ahmed Samy Badran, Hadeel Aboueisha, Atef Akoum, Ayman Elbadawi, Emmanouil S. Brilakis, Fernando Alfonso, Islam Y. Elgendy

**Affiliations:** a Faculty of Medicine, Kafr El-Shaikh University, El-Shaikh, Egypt; b Department of Internal Medicine, American University of Beirut University Medical Center, Beirut, Lebanon; c Faculty of Medicine, Ain-Shams University, Cairo, Egypt; d Faculty of Medical Sciences, University of Groningen, University Medical Center Groningen, Groningen, the Netherlands; e College of Medicine and Health Sciences, United Arab Emirates University, Al Ain, United Arab Emirates; f Department of Medicine, Hennepin Healthcare, Minneapolis, Minnesota, USA; g Department of Cardiology, University of Texas Medical Branch, Galveston, Texas, USA; h Division of Cardiovascular Medicine, Allina Health Heart & Vascular Surgery Center, Abbott Northwestern Hospital, Minneapolis, Minnesota, USA; i Department of Cardiology, Hospital Universitario de La Princesa, IIS-IP, CIBER-CV, Universidad Autónoma de Madrid, Madrid, Spain; j Division of Cardiovascular Medicine, Gill Heart and Vascular Institute, University of Kentucky, Lexington, Kentucky, USA

**Keywords:** complex coronary lesions, drug-eluting stents, intravascular imaging, major adverse cardiovascular events, percutaneous coronary intervention, stent optimization

## Abstract

**Background:**

Intravascular imaging (IVI) guidance improves percutaneous coronary intervention (PCI) outcomes, yet recent randomized controlled trials (RCTs) challenged these findings.

**Objectives:**

This study aimed to evaluate the efficacy and safety of IVI-guided vs angiography-guided PCI in complex coronary lesions, incorporating recent large-scale trials with divergent findings.

**Methods:**

The authors conducted a meta-analysis of RCTs evaluating IVI-guided vs angiography-guided PCI among patients with complex coronary lesions. The primary outcomes were major adverse cardiovascular events (MACE) and cardiac death. Pooled risk ratios (RRs) with 95% CIs were calculated using frequentist random-effects models and complementary Bayesian analyses. The analysis was complemented by several sensitivity and subgroup analyses.

**Results:**

Eighteen RCTs enrolling 18,975 patients were included. IVI guidance was associated with significant reductions in MACE (RR: 0.67; 95% CI: 0.55-0.83), cardiac death (RR: 0.72; 95% CI: 0.53-0.97), myocardial infarction (RR: 0.87; 95% CI: 0.76-0.998), clinically driven target lesion revascularization (RR: 0.72; 95% CI: 0.51-0.999), and stent thrombosis (RR: 0.52; 95% CI: 0.35-0.78). A significant geographic interaction was identified for MACE, with benefit predominantly driven by Asian trial populations (*P*_interaction_ < 0.01). In subgroup analysis, patients achieving imaging-defined stent optimization were associated with lower event rates than those who did not (RR: 0.41; 95% CI: 0.26-0.62).

**Conclusions:**

IVI guidance reduces the risk of adverse events for complex lesions. The benefit was in part driven by achieving imaging-defined stent optimization.

Percutaneous coronary intervention (PCI) with newer-generation drug-eluting stents (DES) remains a cornerstone for the management of patients with acute coronary syndromes (ACSs) and those with chronic coronary syndromes refractory to medical therapy.^1^^,^^2^ Nevertheless, the risk of very late stent failure is not trivial (up to 2%/year) even with newer-generation DES.^3^ The risk of adverse events is even higher among patients with complex coronary lesions (such as unprotected left main, long lesions, in-stent restenosis, bifurcations, and severely calcified lesions).^4^^,^^5^

Conventional coronary angiography has been the standard, but it provides only a 2-dimensional luminogram and is limited in assessing disease morphology, plaque burden, optimal stent implantation, or immediate local complications such as edge dissections.^6^ Intravascular imaging (IVI), including intravascular ultrasound (IVUS) and optical coherence tomography (OCT), provides high-resolution, cross-sectional visualization of the coronary vessel wall.^7^ These modalities enable precise stent optimization, reduce geographic miss, and improve strut apposition.^7^^,^^8^ Randomized controlled trials (RCTs) and meta-analyses demonstrated that IVI reduces major adverse cardiovascular events (MACE) and mortality compared with angiography alone.^9^^,^^10^ The updated American and European guidelines recommend routine IVI for complex PCI.^4^^,^^11^ Despite these recommendations, clinical adoption of intracoronary imaging remains low in Western practice.^12^, ^13^, ^14^ Additionally, trials conducted in East Asian populations drove most of this early clinical benefit, raising questions regarding the generalizability to Western countries.^9^^,^^15^

Recent RCTs in complex lesions have generated conflicting findings. The RENOVATE-COMPLEX-PCI (Intravascular Imaging Guidance Versus Angiography Guidance on Clinical Outcomes After Complex Percutaneous Coronary Intervention) trial demonstrated lower adverse events with IVI guidance.^5^^,^^16^ Conversely, the ILUMIEN IV trial showed that OCT failed to reduce target-vessel failure.^17^ Two large trials, the IVUS-CHIP (Intravascular Ultrasound Guidance for Complex High-Risk Indicated Procedures) and OPTIMAL (Optimization of Left Main PCI With Intravascular Ultrasound) trials, both showed that IVUS guidance did not reduce the primary ischemic endpoints with routine IVUS guidance.^18^^,^^19^ This also highlights uncertainties regarding optimization feasibility in highly resistant lesions.^20^

In this context, we conducted this updated meta-analysis to evaluate the clinical efficacy and safety of IVI-guided (IVUS or OCT) PCI compared with angiography-guided PCI among patients with complex coronary artery lesions.

## Methods

This study was conducted in adherence to the Cochrane Handbook for Systematic Reviews of Interventions and is reported in accordance with the Preferred Reporting Items for Systematic Reviews and Meta-Analyses 2020 statement.^21^^,^^22^ The review protocol was registered on PROSPERO (CRD420261368090).

### Search strategy and screening

A systematic search of MEDLINE (via PubMed), the Cochrane Central Register of Controlled Trials (CENTRAL), Web of Science Core Collection, and Scopus from database inception to April 1, 2026, with no date restrictions applied. The comprehensive search strategy combined both Medical Subject Headings and free-text keywords. The specific Boolean query applied across all databases was: (PCI OR Percutaneous Coronary Intervention) AND (IVUS OR OCT OR IVI OR Intravascular Ultrasound OR Optical Coherence Tomography OR Intravascular imaging OR intracoronary imaging) AND (Calcified Lesion OR Complex lesions OR Complex coronary lesion OR bifurcation OR long coronary lesion OR chronic total occlusions OR ULMCA OR left main coronary artery OR left main). The complete strategy for each database is available in Supplemental Table 1. We additionally searched the reference lists of all included studies and previously published meta-analyses,^10^ and the late-breaking clinical trial proceedings of the ACC (American College of Cardiology) (2026) were manually screened to capture recent data.

All retrieved records were imported into EndNote, and duplicates were removed. Two independent reviewers (A.S.B., M.A.E.) screened titles and abstracts against the prespecified eligibility criteria, followed by full-text review of potentially eligible records. Disagreements at any stage were resolved by a third senior reviewer (I.Y.E.).

### Eligibility criteria

We defined our inclusion criteria using the PICOS framework. We included RCTs enrolling adult patients undergoing PCI with DES for complex coronary artery lesions, defined as high-risk anatomical features, including moderate to severe calcified vessels, bifurcation lesions, long diffuse coronary disease, chronic total occlusions, in-stent restenosis, and left main coronary artery disease. These features were defined in accordance with criteria used in contemporary complex PCI trials and current guidelines,^4^^,^^5^^,^^17^ where long lesions were operationally defined as requiring stent implantation of ≥28 mm in total length;^5^^,^^17^ bifurcation lesions were defined per individual trial eligibility criteria; and moderate-to-severe calcification was defined angiographically or by IVI criteria as reported by each trial.^4^ The intervention of interest was IVI-guided PCI, encompassing both IVUS-guided and OCT-guided strategies. The comparator was standard angiography-guided PCI. Observational studies, case series, editorials, and conference abstracts without full-text availability were excluded. Additionally, trials involving bare-metal stents and studies with <1 year of follow-up were excluded. We did not include studies performing head-to-head comparisons between IVUS-guided and OCT-guided PCI without angiography-guided PCI.

### Data extraction and endpoints

Two independent reviewers (A.S.B., M.A.E.) independently extracted trial-level data using standardized forms. Discrepancies were resolved through discussion. Extracted variables included the study, design, country, sample size, designated treatment arms, follow-up duration, implanted stent type, and the targeted complex lesion type. Detailed baseline clinical, demographic, and angiographic characteristics were extracted. These variables included the number of participants per arm, age, sex distribution, and the prevalence of cardiovascular risk factors (hypertension, dyslipidemia, diabetes mellitus, and current smoking status). We additionally recorded pertinent cardiovascular history (prior myocardial infarction [MI] and prior PCI), mean left ventricular ejection fraction, and baseline angiographic complexity (SYNTAX score).

The primary efficacy outcome was the composite of MACE as defined by the individual trial, as well as cardiac death. Secondary endpoints included all-cause mortality, MI, ischemia or clinically driven target vessel revascularization (TVR), ischemia or clinically driven target lesion revascularization (TLR), stent thrombosis, and target vessel failure (TVF), which was defined as cardiac death, target vessel–related MI, or ischemia-driven TVR. The outcome definitions for each included study are provided in Supplemental Table 2.

### Quality assessment and certainty of evidence

The quality of included studies was independently evaluated by 2 investigators (M.A.E. and A.S.B.). Disagreements were resolved by consensus or by a third author (I.Y.E.). The revised Cochrane Risk of Bias tool (RoB 2)^23^ was used. The certainty of evidence for each outcome was evaluated using the Grading of Recommendations Assessment, Development, and Evaluation framework, incorporating considerations of risk of bias, inconsistency, indirectness, imprecision, and publication bias.^24^

### Statistical analysis

All analyses were performed using RStudio, version 4.4.3 (R Foundation for Statistical Computing). We synthesized aggregate event data utilizing both frequentist and Bayesian analytical frameworks. In the frequentist analysis, pooled risk ratios (RRs) with 95% CIs were computed using a random-effects model with the restricted maximum-likelihood estimator, given the anticipated clinical and methodological heterogeneity across included trials. Statistical significance was defined as *P* < 0.05. Statistical heterogeneity was assessed using the Cochran Q test (significance threshold *P* < 0.10) and quantified with the *I*^2^ statistic. Thresholds of 0% to 49%, 50% to 75%, and >75% were interpreted as low, moderate, and high heterogeneity, respectively. The 95% prediction interval (PI) was reported for all outcomes to communicate the expected range of true effects in future settings.^25^

Bayesian random-effects meta-analyses were performed using the *brms* package in R. A binomial likelihood with a log link was utilized to estimate posterior median RRs and 95% credible intervals. Weakly informative priors were applied for the treatment effect (normal [0, 0.5]) and between-study heterogeneity (Cauchy [0, 0.5]). Posterior distributions were sampled using Markov chain Monte Carlo. Additionally, we studied the posterior probabilities of the 4 thresholds: any harm (RR >1), any benefit (RR <1), a 10% risk reduction (RR <0.90), and a 20% risk reduction (RR <0.80).

Subgroup and sensitivity analyses were conducted for the primary outcomes based on: 1) imaging modality (IVUS vs OCT); 2) geographic region (Asian vs Western populations); and 3) sensitivity analysis excluding first-generation DES. We conducted subgroup analyses by lesion type: unprotected left main coronary artery disease, chronic total occlusions, bifurcation lesions, moderate-to-severe calcified lesions, long coronary lesions, multivessel disease, and in-stent restenosis, and clinical presentation (ACS vs non-ACS). Differences in subgroup-specific treatment effects were assessed using Cochran’s Q test within the frequentist framework. We prespecified a secondary within-arm analysis restricted to the imaging-guided cohort to compare optimal vs suboptimal post-PCI intracoronary imaging results as defined per each study.

A prespecified sensitivity analysis was conducted by sequentially excluding one study at a time (leave-one-out analysis) to assess the robustness of pooled estimates to individual trial influence. Publication bias was evaluated using visual inspection of funnel plots and Egger's regression test (significance threshold *P* < 0.10). The trim-and-fill method was applied to estimate the number of potentially suppressed studies and recalculate adjusted pooled estimates.

Individual patient data (IPD) were reconstructed from published Kaplan-Meier curves using the method described by Guyot et al^26^ Kaplan-Meier curves were digitized with WebPlotDigitizer, and the extracted coordinates were combined with the reported numbers of patients, events, and numbers at risk at prespecified time points. IPD reconstruction was performed using the IPDfromKM package in R. The reconstructed data sets were validated by comparing the reconstructed survival estimates with the published Kaplan-Meier curves and reported summary data. Study-specific HRs were subsequently estimated by fitting Cox proportional hazards regression models to the reconstructed IPD.

## Results

### Search results and study selection

The initial systematic database search yielded 8,921 citations. After removing 2,789 duplicates, 6,132 citations underwent title and abstract screening. Of these, 48 reports were retrieved for full-text eligibility assessment. Finally, 18 RCTs met the inclusion criteria for our systematic review (Figure 1).

**Figure 1:**
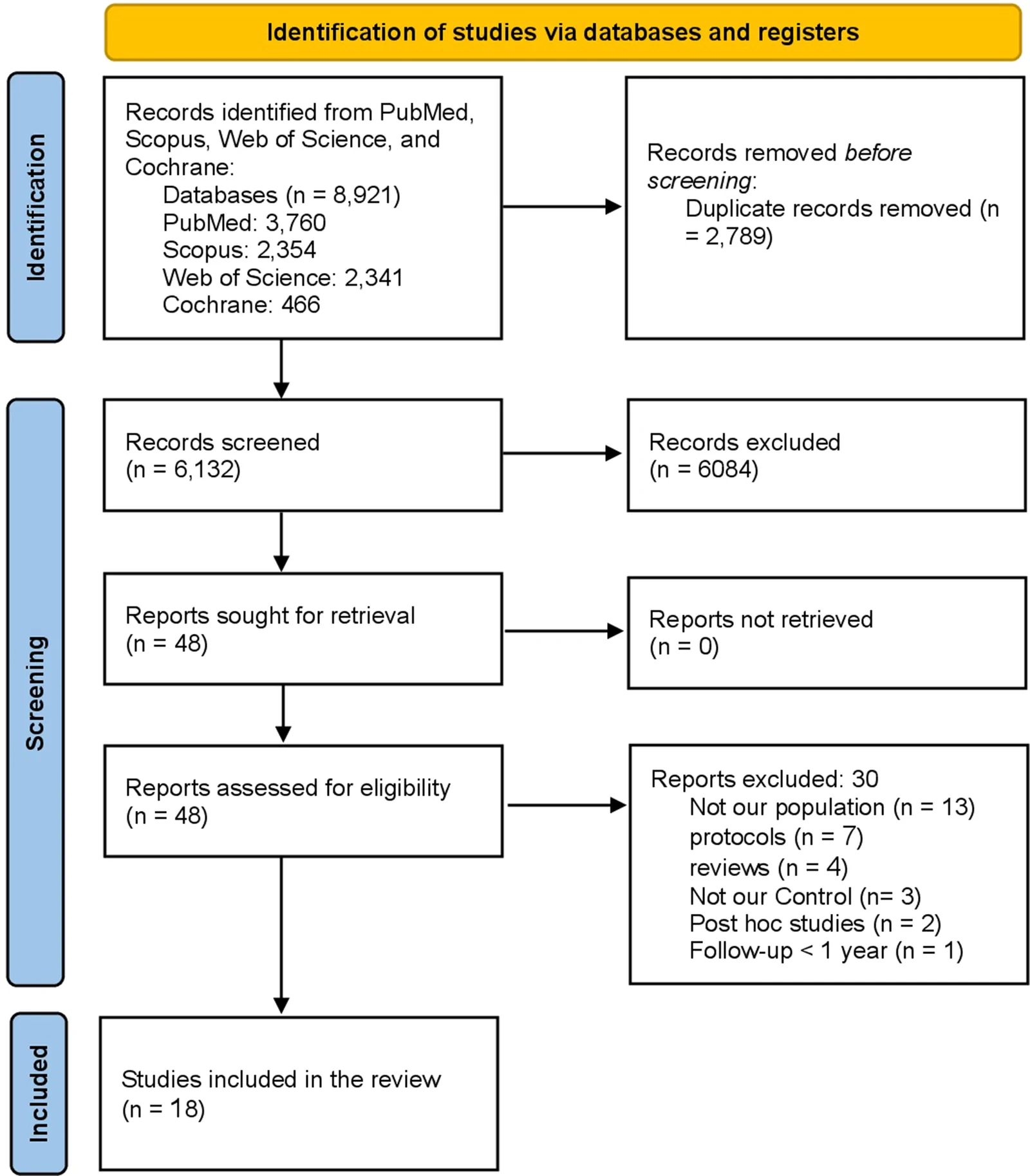
Preferred Reporting Items for Systematic Reviews and Meta-Analyses Flowchart Illustrating the Process of Study Selection WOS = Web of Science.

### Characteristics of included studies

A total of 18,975 patients from 18 RCTs^5^^,^^7^^,^^16^, ^17^, ^18^, ^19^^,^^27^, ^28^, ^29^, ^30^, ^31^, ^32^, ^33^, ^34^, ^35^, ^36^, ^37^, ^38^, ^39^, ^40^, ^41^ were included in this systematic review. A total of 6,829 patients were assigned to the IVUS group, 2,914 to the OCT group, and 9,231 to the angiography group. The study population had a weighted mean age of 65.1 years, and 75.8% of patients were male. The weighted mean baseline left ventricular ejection fraction was 57.9%, and the weighted mean follow-up duration across trials was 26.3 months. Comprehensive baseline and study-level characteristics are detailed in Table 1, Table 2, and Supplemental Table 3.

**Table 1:** Summary of Included Studies

First Author, Year, Study	Study Design	Country	Sample Size, Arms	Follow-Up duration	Stent Type	Lesion Type
Testa et al,^18^ 2026, OPTIMAL	Investigator-initiated, international, multicenter, open-label, superiority RCT	Italy, Spain, and the UK.	806 (401 for IVUS vs 405 for angiography)	2.9 y (median)	Everolimus-eluting platinum–chromium stents	Unprotected LMCA disease
Gao et al,^27^ 2026, DKCRUSH VIII	Multicenter, open-label, controlled, superiority RCT	China	556 (278 for IVUS vs 278 for angiography)	1 y	Standard DK crush technique with second-generation DES	Complex coronary bifurcation lesion
Diletti et al,^19^ 2026, IVUS-CHIP	Investigator-initiated, multicenter, international, open-label, event-driven, controlled RCT	7 European countries	2020 (1,010 for IVUS vs 1,010 for angiography)	19 mo (median)	Everolimus-eluting platinum–chromium stents	Complex high-risk PCI, defined as angiographic severe calcifications, ostial lesions, true bifurcation lesions, LMCA lesions, CTOs, ISR, or lesions more than 28 mm in length.
Ali et al,^17^ 2023, ILUMIEN IV	Multicenter, prospective, large-scale, single-blind, RCT	United States	2,487 (1,233 for OCT vs 1,254 for angiography)	2 y	Second-generation DES XIENCE (Abbott)	Recent MI, >28-mm lesion, bifurcation, severely calcified lesion, CTO, diffuse or multifocal ISR
Bendary et al,^28^ 2024	2-center RCT	Egypt	181 (90 for IVUS vs 91 for angiography)	1 y	Second-generation DES	Unprotected LMCA lesions
Chieffo et al,^29^ 2013, AVIO	Multicenter, international, open-label, investigator-driven RCT	Italy, France, Spain, Greece, Latvia, Finland, the UK	284 (142 for IVUS vs 142 for angiography)	2 y	Second-generation DES	Complex lesions: bifurcations, long lesions, CTOs, small vessels^c^
Gao et al,^30^ 2021; Zhang et al,^31^ 2018, ULTIMATE^,^^a^	Multicenter, prospective, RCT	China	1,448 (724 for IVUS vs 724 for angiography)	3 y	Second DES	CTOs, bifurcation lesion, calcified lesions, long lesions, MVD
Holm et al,^32^ 2023, OCTOBER	Multicenter, open-label, controlled, superiority RCT	European countries	1,201 (600 for OCT vs 601 for angiography)	2 y	Second-generation DES (Xience everolimus-eluting stent (Abbott))	Coronary bifurcation lesion
Hong et al,^7^ 2015; Hong et al,^33^ 2020, IVUS-XPL	Multicenter, prospective, RCT	Korea	1,400 (700 for IVUS vs 700 for angiography)	5.3 y	Second-generation DES (Everolimus-eluting)	Long coronary lesions (implanted stent ≥28 mm in length)
Jakabčin et al,^34^ 2010, HOME DES IVUS	Single-center, prospective, RCT	Czech Republic	210 (105 for IVUS vs 105 for angiography)	18 mo	First-generation DES (TAXUS and CYPHER)	Complex coronary lesions: proximal LAD, LMCA, small vessels, lesion length >20 mm, ISR
Kim et al,^35^ 2013, RESET	Multicenter, prospective, open-label RCT	South Korea	543 (269 for IVUS vs 274 for angiography)	1 y	DES (Zotarolimus-eluting stents, Everolimus-eluting stents)	Long coronary artery stenoses
Kim et al,^36^ 2015, CTO-IVUS	Multicenter, prospective, open-label RCT	Korea	402 (201 for IVUS vs 201 for angiography)	1 y	Second- and third-generation DES (Resolute zotarolimus-eluting stents, Nobori biolimus-eluting stents)	CTO lesions
Lee et al,^5^ 2023; Lee et al,^16^ 2026, RENOVATE-COMPLEX-PCI	Multicenter, investigator-initiated, prospective, open-label RCT	South Korea	1,639 (1,092 for IVI vs 547 for angiography)	5 y	Second-generation DES (biodegradable or biocompatible polymer-coated everolimus-eluting stents)	Complex coronary artery lesions: true bifurcation lesions, chronic total occlusion; unprotected LMCA; long coronary-artery lesions; multivessel PCI, lesions necessitate multiple stents; ISR; severely calcified lesion; or ostial lesions of a major epicardial coronary artery.
Li et al,^41^ 2024, IVUS-ACS^b^	Multicenter, two-stage, masked RCT	China, Italy, Pakistan, and the UK	3,505 (1753 for IVUS vs 1752 for angiography)	1 y	Second DES	Multivessel disease
Liu et al,^40^ 2019	Single-center, open-label RCT	China	336 (167 for IVUS vs 169 for ICA)	1 y	DES	LMCA lesion
Tan et al,^37^ 2015	RCT	China	123 (61 for IVUS vs 62 for Angiography)	2 y	Sirolimus-eluting DES	ULMCA stenosis
Tian et al,^38^ 2015, AIR-CTO	Prospective, Two-center RCT	China	230 (115 for IVUS vs 115 for Angiography)	2 y	First-generation DES	CTO lesions
Hong et al,^39^ 2024, OCCUPI	Investigator-initiated, multicenter, open-label, superiority RCT	South Korea	1,604 (803 for OCT vs 801 for angiography)	1 y	Second-generation DES (everolimus-eluting stents)	Acute MI, CTO, long lesion, calcified lesion, bifurcation lesion, unprotected LMCA disease, small vessel disease,^c^ intracoronary thrombus visible on angiography, stent thrombosis, ISR, or bypass graft lesion.

AVIO, Angiography Versus IVUS Optimization; CAD = coronary artery disease; CTO = chronic total occlusion; CTO-IVUS, Chronic Total Occlusion Intervention With Drug-Eluting Stents Guided by IVUS; DES, drug-eluting stent; DKCRUSH VIII, IVUS-Guided DK Crush Stenting Technique for Patients With Complex Bifurcation Lesions; ICA = invasive coronary angiography; ISR = in-stent restenosis; IVI = intravascular imaging; IVUS = intravascular ultrasound; IVUS-ACS, Intravascular Ultrasound in Acute Coronary Syndrome; IVUS-CHIP, Intravascular Ultrasound Guidance for Complex High-Risk Indicated Procedures; IVUS-XPL, Impact of Intravascular Ultrasound Guidance on Outcomes of Xience Prime Stents in Long Lesions; LAD = left anterior descending artery; LMCA = left main coronary artery; MVD = multivessel disease; OCCUPI, Optical Coherence Tomography-Guided Coronary Intervention in Patients With Complex Lesions; OCT = optical coherence tomography; OCTOBER, Optical Coherence Tomography Optimized Bifurcation Event Reduction; OFDI = optical frequency domain imaging; OPTIMAL, Optimization of Left Main PCI With Intravascular Ultrasound; PCI = percutaneous coronary intervention; RENOVATE-COMPLEX-PCI, Intravascular Imaging Guidance Versus Angiography Guidance on Clinical Outcomes After Complex Percutaneous Coronary Intervention; RESET, Real Safety and Efficacy of a 3-Month Dual Antiplatelet Therapy Following Zotarolimus-Eluting Stents Implantation; ULMCA = unprotected left main coronary artery; ULTIMATE, Intravascular Ultrasound Guided Drug-Eluting Stents Implantation in “All-Comers” Coronary Lesions.

a We included only patients with complex CAD in the subgroup analysis and reported outcomes related to stent optimization.

b This study included only patients with acute coronary syndromes and reported subgroup analyses limited to multivessel lesions, as well as data on stent optimization.

c Small vessel diseases (vessel diameter <2.5 mm).

**Table 2:** Baseline Characteristics of Included Studies

First Author, Year, Study	Arms	N	Age	Male	Hypertension	Dyslipidemia	Diabetes	Current Smoker	Prior MI	Prior PCI	LVEF, %	SYNTAX Score	Optimal Post-PCI IVUS Rates, %
Testa et al,^18^ 2026, OPTIMAL	IVUS-guided	401	71.1 ± 11.0	315 ± 78.6	318 ± 79.5	296 ± 74.0	147 ± 36.6	52 ± 13.0	100 ± 25.0	105 ± 26.2	53.2 ± 11.1	30.1 ± 13.0	NA
	Angiography-guided	405	71.7 ± 10.4	317 ± 78.3	321 ± 79.3	315 ± 77.8	133 ± 32.8	61 ± 15.1	90 ± 22.2	100 ± 24.7	53.4 ± 10.4	29.3 ± 12.1	NA
Gao et al,^27^ 2026, DKCRUSH VIII	IVUS-guided	278	66 ± 11.2	215 ± 77.6	190 ± 68.6	120 ± 43.3	89 ± 32.1	78 ± 28.2	42 ± 15.2	82 ± 29.6	61.67 ± 5.22	60.9% had a SYNTAX score ≥32	75.4%
	Angiography-guided	278	65.33 ± 10.4	215 ± 77.3	171 ± 61.5	127 ± 45.7	95 ± 34.2	75 ± 27.0	48 ± 17.3	96 ± 34.5	61.67 ± 5.22		
Diletti et al,^19^ 2026, IVUS-CHIP	IVUS-guided	1,010	69.5 ± 10.3	804 ± 79.6	762 ± 75.4	719 ± 71.2	310 ± 30.7	181 ± 17.9	297 ± 29.4	472 ± 46.7	52.6 ± 11.0	25.4 ± 14.4	53.3%
	Angiography-guided	1,009	69.1 ± 10.3	800 ± 79.3	784 ± 77.7	708 ± 70.2	322 ± 31.9	170 ± 16.8	318 ± 31.5	481 ± 47.7	53.3 ± 11.5	24.9 ± 14.0	
Ali et al,^17^ 2023, ILUMIEN IV	OCT-guided	1,233	65.5 ± 10.5	968 ± 78.5	880 ± 71.4	808 ± 65.5	523 ± 42.4	242 ± 19.6	252 ± 20.4	160 ± 13.3	55.2 ± 8.6	NA	49.8%
	Angiography-guided	1,254	65.7 ± 10.3	956 ± 76.2	928 ± 74	860 ± 68.6	521 ± 41.5	247 ± 19.7	303 ± 24.2	166 ± 13.4	55.2 ± 8.7	NA	
Bendary et al,^28^ 2024	IVUS-guided	90	63 ± 9	66 ± 73.3	68 ± 75.6	67 ± 74.4	56 ± 62.2	52 ± 57.8	16 ± 17.8	33 ± 36.7	55 ± 9	27.6 ± 6	NA
	Angiography-guided	91	61 ± 9	63 ± 69.2	61 ± 67	58 ± 63.7	63 ± 69.2	50 ± 54.9	13 ± 14.3	26 ± 28.6	54 ± 8	26.4 ± 6.8	NA
Chieffo et al,^29^ 2013, AVIO	IVUS-guided	142	63.9 ± 10.1	117 ± 82.4	100 ± 70.4	100 ± 70.4	34 ± 23.9	49 ± 34.5	N.A	N.A	55.3 ± 8.5	NA	48.1%
	Angiography-guided	142	63.6 ± 11	109 ± 76.8	95 ± 66.9	109 ± 76.8	38 ± 26.8	44 ± 31	N.A	N.A	55.9 ± 8.6	NA	
Gao et al,^30^ 2021; Zhang et al,^31^ 2018, ULTIMATE	IVUS-guided	724	65.2 ± 10.9	535 ± 73.9	512 ± 70.7	389 ± 53.7	217 ± 30	253 ± 34.9	67 ± 9.3	126 ± 17.4	60.9 ± 7.9	NA	53%
	Angiography-guided	724	65.9 ± 9.8	530 ± 73.2	521 ± 72	400 ± 55.2	226 ± 31.2	228 ± 31.5	86 ± 11.9	144 ± 19.9	60.3 ± 9.3	NA	
Holm et al,^32^ 2023, OCTOBER	OCT-guided	600	66.4 ± 10.5	473 ± 78.8	422 ± 70.3	NA	103 ± 17.2	N.A	170 ± 28.3	244 ± 40.7	56 ± 7.4	NA	71.1%
	Angiography-guided	601	66.2 ± 9.9	475 ± 79	448 ± 74.5	NA	97 ± 16.1	N.A	180 ± 30	257 ± 42.8	59 ± 14.1	NA	
Hong et al,^7^ 2015; Hong et al,^33^ 2020, IVUS-XPL	IVUS-guided	700	64 ± 9	483 ± 69	454 ± 65	471 ± 67	250 ± 36	155 ± 22	34 ± 5	76 ± 11	62.9 ± 9.8	NA	54%
	Angiography-guided	700	64 ± 9	481 ± 69	444 ± 63	458 ± 65	256 ± 37	181 ± 26	29 ± 4	69 ± 10	62.4 ± 10.2	NA	
Jakabcin et al,^34^ 2010	Angiography-guided	105	60.2 ± 11	75 ± 71	75 ± 71	NA	47 ± 45	37 ± 35	34 ± 32	15 ± 14	N.A	NA	NA
	IVUS-guided	105	59.4 ± 13	77 ± 73	70 ± 67	NA	44 ± 42	42 ± 40	39 ± 37	18 ± 17	N.A	NA	NA
Kim et al,^35^ 2013, RESET	IVUS-guided	269	62.8 ± 9.3	177 ± 65.8	165 ± 61.3	165 ± 61.3	85 ± 31.6	58 ± 21.6	3 ± 1.1	N.A	55.3 ± 23.9	NA	NA
	Angiography-guided	274	64.3 ± 8.7	150 ± 54.7	178 ± 65.8	165 ± 61.7	82 ± 29.9	47 ± 17.2	8 ± 2.9	N.A	54 ± 25	NA	NA
Kim et al,^36^ 2015, CTO-IVUS	IVUS-guided	201	61 ± 11.1	162 ± 80.6	126 ± 62.7	N.A	70 ± 34.8	71 ± 35.3	16 ± 8	31 ± 15.4	56.9 ± 13.1	NA	NA
	Angiography-guided	201	61.4 ± 10.1	162 ± 80.6	128 ± 63.7	N.A	68 ± 33.8	69 ± 34.3	16 ± 8	32 ± 15.9	56.7 ± 11.4	NA	NA
Lee et al,^5^ 2023; Lee et al,^16^ 2026, RENOVATE-COMPLEX-PCI	IVUS-guided	1,092	65.3 ± 10.3	869 ± 79.6	682 ± 62.5	560 ± 51.3	394 ± 36.1	212 ± 19.4	75 ± 6.9	268 ± 24.5	58.4 ± 11.9	NA	45.4%
	Angiography-guided	547	66.0 ± 10	431 ± 78.8	323 ± 59	280 ± 51.2	223 ± 40.8	95 ± 17.4	42 ± 7.7	127 ± 23.2	59.3 ± 11	NA	
Li et al,^41^ 2024, IVUS-ACS	IVUS-guided	1,753	61.6 ± 11.1	1,285 ± 73.3	NA	1,103 ± 62.9	1,187 ± 67.7	554 ± 31.6	499 ± 28.5	111 ± 6.3	60.7 ± 7.4	NA	79.9%
	Angiography-guided	1,752	62 ± 11.1	1,299 ± 74.1	NA	1,089 ± 62.2	1,222 ± 69.8	551 ± 31.5	487 ± 27.8	106 ± 6.1	60.7 ± 7.4	NA	
Liu et al,^40^ 2019	IVUS-guided	167	65.3 ± 10.6	106 ± 63.5	116 ± 69.5	63 ± 37.7	56 ± 33.5	62 ± 37.1	29 ± 17.4	33 ± 19.8	55.6 ± 11.7	28.1 ± 7.5	NA
	ICA-guided	169	64.9 ± 11.2	108 ± 63.9	122 ± 72.2	64 ± 37.9	52 ± 30.8	60 ± 35.5	24 ± 14.2	28 ± 16.6	58.4 ± 10.5	30.2 ± 10.1	NA
Tan et al,^37^ 2015	Angiography-guided	62	75.9 ± 3.5	43 ± 69.4	29 ± 46.8	NA	18 ± 29.5	29 ± 46.8	13 ± 21	NA	53.3 ± 7.1	NA	NA
	IVUS-guided	61	76.5 ± 5	38 ± 62.3	25 ± 41	NA	21 ± 34.4	27 ± 44.3	10 ± 16.4	NA	55.3 ± 5	NA	NA
Tian et al,^38^ 2015, AIR-CTO	Angiography-guided	115	66 ± 11	92 ± 80	81 ± 70.4	32 ± 27.8	31 ± 27	45 ± 39.1	35 ± 30.4	24 ± 20.9	56 ± 12	NA	68%
	IVUS-guided	115	67 ± 10	102 ± 88.7	86 ± 74.8	25 ± 21.9	34 ± 29.6	45 ± 39.1	24 ± 20.9	23 ± 20	55 ± 11	NA	91%
Hong et al,^39^ 2024, OCCUPI	OCT-guided	803	63.7 ± 9.7	646 ± 80	466 ± 58	684 ± 85	261 ± 33	149 ± 19	40 ± 5	171 ± 21	59.5 ± 8.8	NA	NA
	Angiography-guided	801	64 ± 8.9	644 ± 80	451 ± 56	661 ± 83	262 ± 33	158 ± 20	42 ± 5	159 ± 20	59.7 ± 10.1	NA	NA

Values are mean ± SD or n (%), unless otherwise indicated. LVEF = left ventricular ejection fraction; NA = not available; other abbreviations as in Table 1.

### Quality assessment and publication bias

The risk of bias was assessed using RoB2, which highlighted an overall low risk of bias for 16 trials, while 2 studies were noted for overall some concerns (Supplemental Figure 1). According to the Grading of Recommendations Assessment, Development, and Evaluation framework, the certainty of evidence was rated as high for stent thrombosis. The certainty was moderate for MACE, cardiac death, all-cause mortality, MI, ischemia, or clinically driven TLR, while TVF was ranked as low and ischemia- or clinically driven TVR as very low (Supplemental Table 4). Notably, most outcomes exhibited evidence of publication bias. Following adjustment using the trim-and-fill method, the previously significant findings were no longer statistically significant for most outcomes (Supplemental Table 5 and Supplemental Figures 2-5).

### Meta-analysis

#### Primary outcomes

A total of 7,320 patients from 12 RCTs were included in the pooled analysis of MACE. The incidence of MACE was lower with IVI compared with angiography. The use of IVI was associated with a significant reduction in the risk of MACE compared with angiography (RR: 0.67; 95% CI: 0.55-0.83; *P* < 0.01); however, the 95% PI was wide (0.38-1.20). The analysis showed moderate heterogeneity (*I*^2^ = 62.0%; *P* < 0.01) (Figure 2). This was consistent with the reconstructed IPD from 6 studies.^7^^,^^18^^,^^33^^,^^36^^,^^37^^,^^40^ The IVI significantly reduced the MACE risk by 25% (HR: 0.75; 95% CI: 0.65-0.88; *P* < 0.01) over 5 years (Figure 3). In subgroup analyses, the benefits of IVI remained consistent regardless of imaging modality (IVUS vs OCT). Notably, a significant geographical interaction was observed: the reduction in MACE was maintained in Asian populations, whereas no significant difference was observed in Western populations (*P*_interaction_ < 0.01). Sensitivity analysis for MACE excluding studies involving first-generation DES was consistent with the primary finding (Supplemental Figure 6-8).

**Figure 2:**
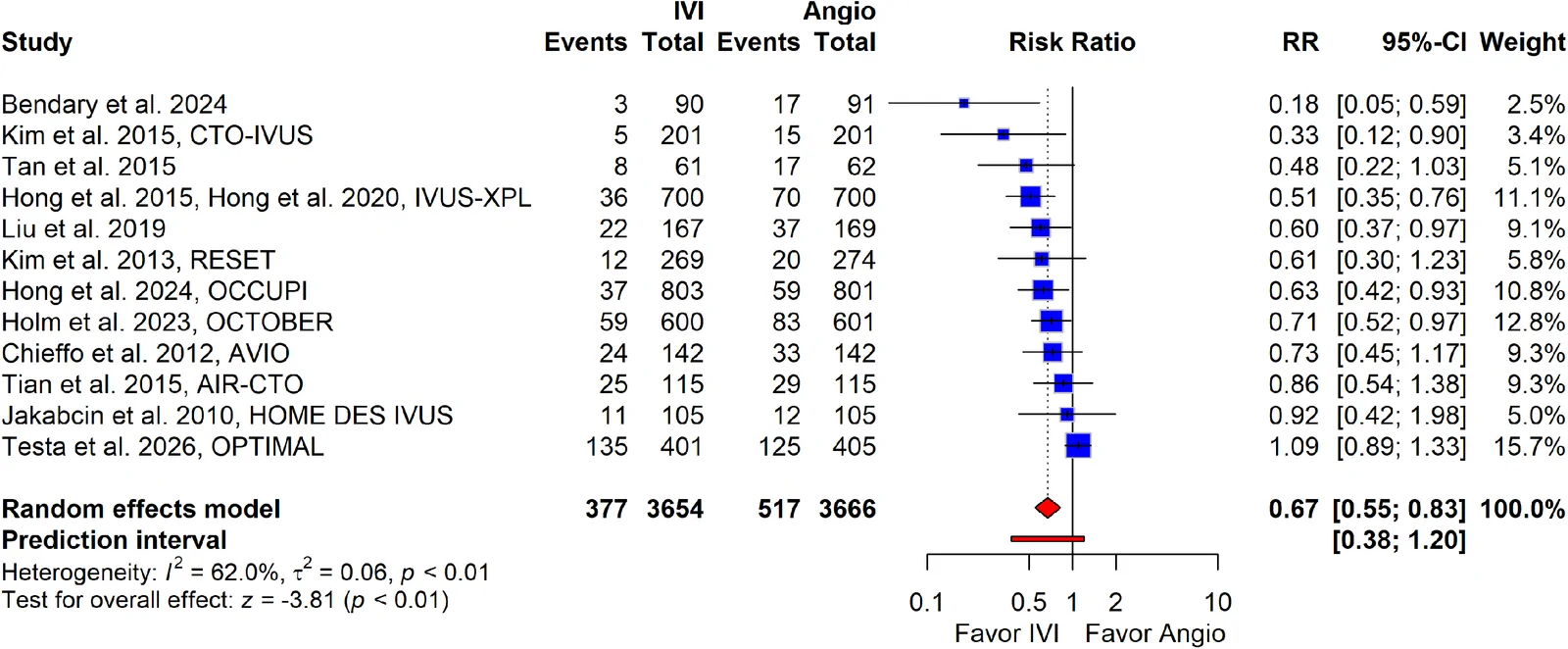
Forest Plot for MACE IVI = intravascular imaging; MACE = major adverse cardiovascular events; RR = risk ratio.

**Figure 3:**
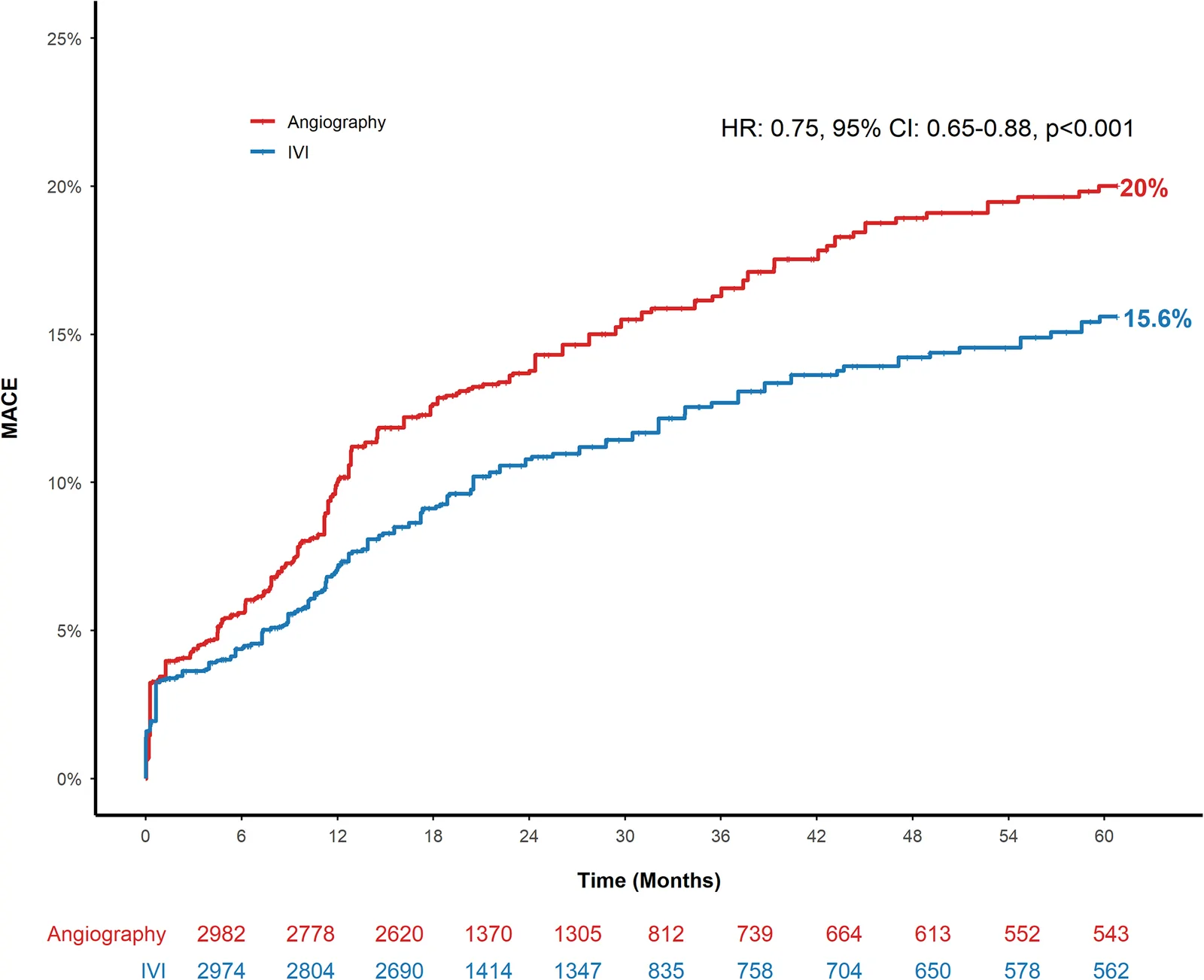
Reconstructed Individual Patient Data Kaplan-Meier Curves for MACE Abbreviations as in Figure 2.

Regarding cardiac death, 14 RCTs, including 13,608 patients, were pooled in the analysis. IVI was associated with a significantly lower risk of cardiac death compared with angiography (RR: 0.72; 95% CI: 0.53-0.97; *P* = 0.03). The 95% PI was wide (0.36-1.41). The heterogeneity was low (*I*^2^ = 26.1%; *P* = 0.17) (Figure 4, Central Illustration). In subgroup analyses, OCT and Asian populations were consistent with the primary analysis, whereas IVUS and Western populations did not show statistically significant differences. The sensitivity analysis excluding studies involving first-generation DES yielded results consistent with the primary analysis (Supplemental Figures 9 to 11).

**Figure 4:**
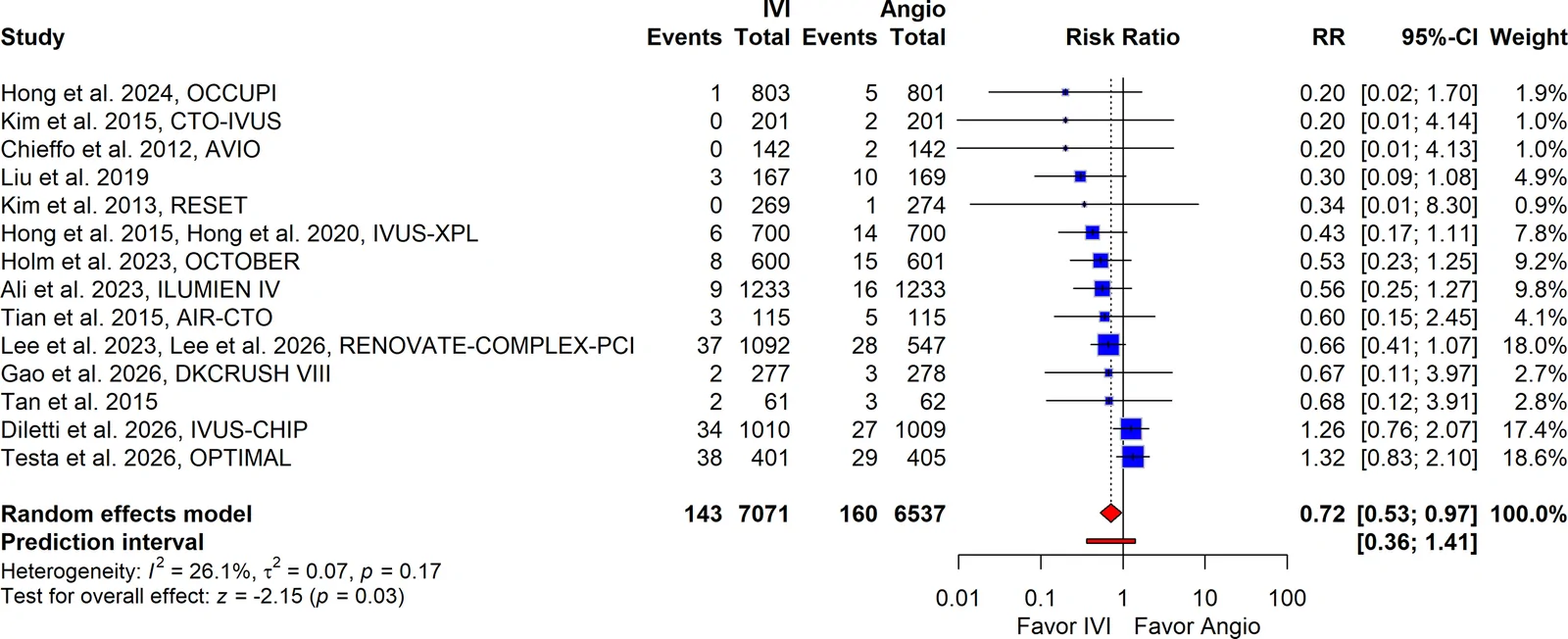
Forest Plot for Cardiac Death Abbreviations as in Figure 2.

**Central Illustration:**
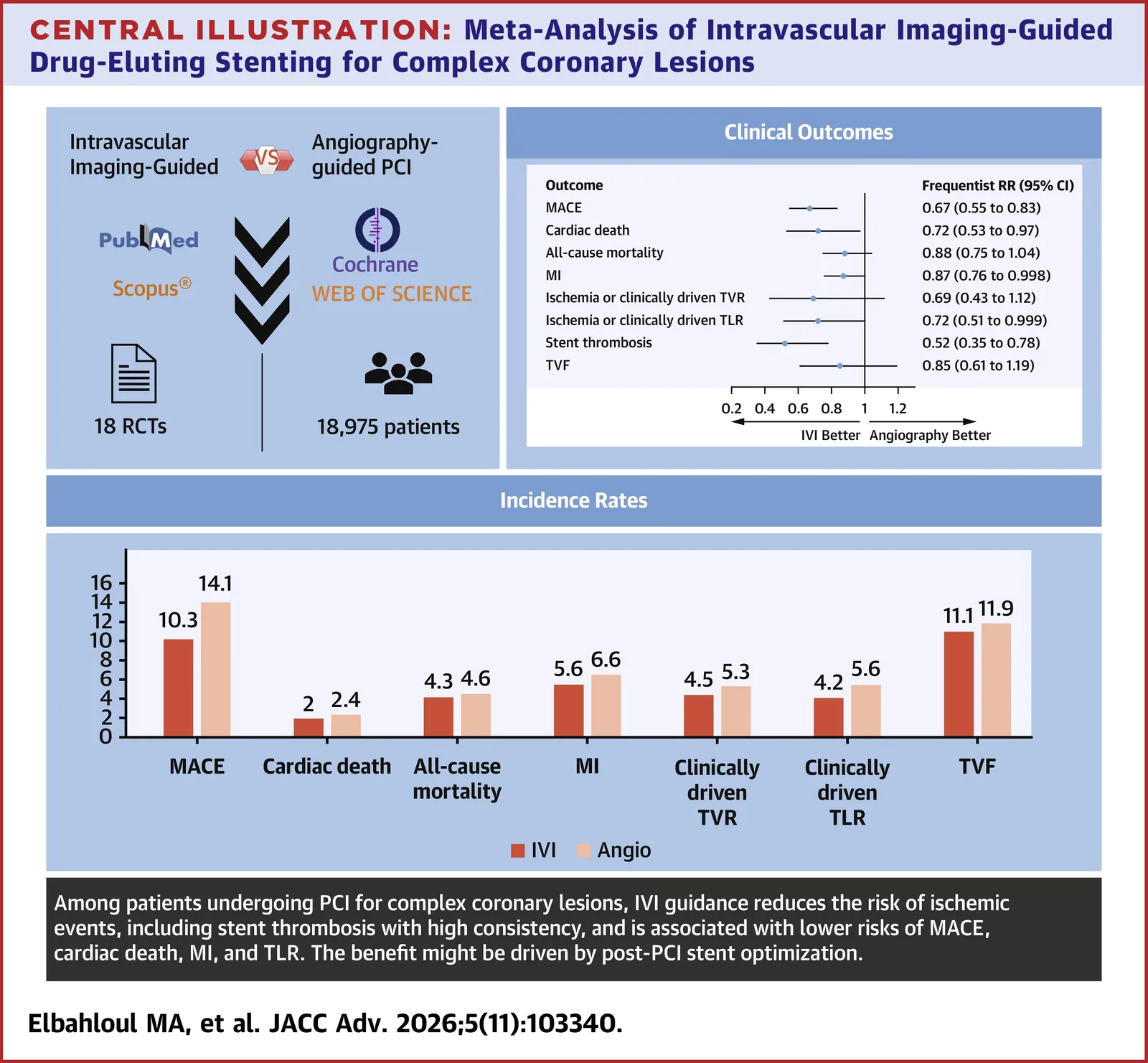
Meta-Analysis of Intravascular Imaging-Guided Drug-Eluting Stenting for Complex Coronary Lesions Among patients undergoing PCI for complex coronary lesions, intravascular imaging guidance reduces the risk of ischemic events, including stent thrombosis with high consistency, and is associated with lower risks of MACE, cardiac death, MI, and target lesion revascularization. The benefit might be driven by post-PCI stent optimization. IVI = intravascular imaging; MACE = major adverse cardiovascular events; MI = myocardial infarction; PCI = percutaneous coronary intervention; RCT = randomized controlled trial; RR = risk ratio; TLR = target lesion revascularization; TVF = target vessel failure; TVR = target vessel revascularization.

#### Secondary outcomes

Pooled results are detailed in Table 3. IVI was associated with a significant reduction in the risk of MI (RR: 0.87; 95% CI: 0.76-0.998; *P* = 0.046; *I*^2^ = 0%), ischemia or clinically driven TLR (RR: 0.72; 95% CI: 0.51-0.999; *P* = 0.049; *I*^2^ = 52.7%), and stent thrombosis (RR: 0.52; 95% CI: 0.35-0.78; *P* < 0.01; *I*^2^ = 0.7%). However, no significant difference was observed between IVI and angiography in all-cause mortality (RR: 0.88; 95% CI: 0.75-1.04; *P* = 0.142; *I*^2^ = 0), ischemia or clinically driven TVR (RR: 0.69; 95% CI: 0.43-1.12; *P* = 0.13; *I*^2^ = 76.4%), and TVF (RR: 0.85; 95% CI: 0.61-1.19; *P* = 0.34; *I*^2^ = 80.4%) (Supplemental Figures 12-17).

**Table 3:** Summary of Outcomes Using Frequentist-Based and Bayesian Analyses

	Participants (Studies) Follow-Up	Study Event Rates (%)		Risk Difference with IVI	Frequentist RR (95% CI)	Bayesian RR (95% CrI)	Overall Certainty of Evidence
		With Angiography	With IVI				
MACE	7,320 (12 RCTs)	517/3,666 (14.1%)	377/3,654 (10.3%)	47 fewer per 1,000 (from 63 fewer to 24 fewer)	0.67 (0.55-0.83)	0.76 (0.67-0.86)	Moderate
Cardiac death	13,608 (14 RCTs)	160/6,537 (2.4%)	143/7,071 (2.0%)	7 fewer per 1,000 (from 12 fewer to 1 fewer)	0.72 (0.53-0.97)	0.80 (0.64-0.98)	Moderate
All-cause mortality	11,856 (12 RCTs)	258/5,660 (4.6%)	267/6,196 (4.3%)	5 fewer per 1,000 (from 11 fewer to 2 more)	0.88 (0.75-1.04)	0.89 (0.75-1.04)	Moderate
MI	12,599 (15 RCTs)	401/6,033 (6.6%)	370/6,566 (5.6%)	9 fewer per 1,000 (from 17 fewer to 1 fewer)	0.87 (0.76-0.998)	0.87 (0.75-0.99)	Moderate
Ischemia or clinically driven TVR	7,845 (5 RCTs)	209/3,922 (5.3%)	178/3,923 (4.5%)	17 fewer per 1,000 (from 30 fewer to 6 more)	0.69 (0.43-1.12)	0.87 (0.71-1.05)	Low
Ischemia or clinically driven TLR	6,428 (5 RCTs)	181/3,217 (5.6%)	135/3,211 (4.2%)	16 fewer per 1,000 (from 28 fewer to 1 fewer)	0.72 (0.51-0.999)	0.76 (0.61-0.94)	Moderate
Stent thrombosis	11,988 (13 RCTs)	86/5,728 (1.5%)	45/6,260 (0.7%)	7 fewer per 1,000 (from 10 fewer to 3 fewer)	0.52 (0.35-0.78)	0.56 (0.40-0.76)	High
TVF	7,485 (5 RCTs)	413/3,472 (11.9%)	444/4,013 (11.1%)	18 fewer per 1,000 (from 46 fewer to 23 more)	0.85 (0.61-1.19)	0.94 (0.83-1.06)	Moderate

CrIs = credible intervals; MACE = major adverse cardiovascular events; RR = risk ratio; TLR = target lesion revascularization; TVF = target vessel failure; TVR = target vessel revascularization; other abbreviations as in Table 1.

### Bayesian analysis

The results were consistent with the frequentist approach analysis (Table 3, Supplemental Figures 18 and 19). Furthermore, the Bayesian posterior probabilities for varying thresholds of risk reduction across all outcomes are detailed in Supplemental Table 6.

### Subgroup analysis by lesion type for composite of MACE or TVF

Pooled results are detailed in Supplemental Table 7. The use of IVI was associated with a significant reduction in left main coronary artery disease, long coronary lesions, multivessel disease, and ACS.

In a separate analysis of 6 studies evaluating stent optimization, the definition used in each study is provided in Supplemental Table 2. Optimal PCI was associated with a lower risk of the composite outcome of MACE or TVF compared with suboptimal PCI (RR: 0.41; 95% CI: 0.26-0.62; *P* < 0.01; *I*^2^ = 62.7%) (Supplemental Figure 20).

### Sensitivity analysis

A leave-one-out sensitivity analysis was performed for all outcomes (Supplemental Figures 21-28). The results for MACE, all-cause mortality, stent thrombosis, and TVF remained consistent with the primary analysis, indicating that no single study disproportionately influenced these findings. In contrast, the results for cardiac death, MI, ischemia, or clinically driven TVR, and clinically driven TLR were not consistent and were influenced by individual studies.

## Discussion

In this meta-analysis of 18 RCTs with 18,975 patients undergoing PCI for complex coronary lesions, IVI guidance was associated with lower risks of MACE, cardiac death, MI, clinically driven TLR, and stent thrombosis compared with angiography-guided PCI. The most robust finding was the reduction in stent thrombosis. Overall, these findings support IVI as a beneficial strategy for complex PCI, though its clinical benefits might be tied to the quality of postprocedural implementation. Bayesian posterior distributions demonstrated a high probability of clinical benefit in reducing MACE. These reductions were primarily driven by lower incidences of MI, stent thrombosis, and TLR. Notably, mitigating the need for TLR is an important clinical objective, as subsequent revascularization has been shown to be an independent predictor of long-term mortality.^42^

Among all outcomes evaluated in this analysis, stent thrombosis provides the clearest justification for routine IVI use in complex PCI. Our meta-analysis found a significant reduction in the risk of stent thrombosis with a high degree of certainty. Stent thrombosis remains a concern even with the widespread use of potent P2Y12 inhibitors and newer-generation DES.^43^ Stent thrombosis is often related to underexpansion, malapposition, or edge dissection, and IVI could help to identify these complications.^44^ Drug-coated balloons represent an emerging strategy that avoids permanent metallic implantation and may reduce the thrombotic substrate inherent to stent-based PCI.^44^ Data on IVI-guided drug-coated balloon strategy in complex coronary lesions are currently lacking and represent an important direction for future investigation.^44^ The secondary endpoints showed a favorable but less consistent pattern. IVI was associated with lower MI and TLR, whereas all-cause mortality, TVR, and TVF did not reach statistical significance. This distinction is clinically relevant because IVI appeared to have a clearer effect on lesion-level failure than on broader vessel-level or composite endpoints such as TVF, which were more heterogeneous across trials. The secondary outcomes support a broader directional benefit of IVI but reinforce that stent thrombosis was the most internally consistent endpoint in this analysis.

In our geography-based analysis, the benefit was observed mainly in Asian trials. We speculate that IVI adoption differs substantially across regions, with higher use in East Asian catheterization laboratories and lower or more variable use in Western practice.^13^^,^^14^ In centers where imaging is part of everyday workflow, such as in Asian countries, image acquisition, interpretation, and optimization may become more standardized. Additionally, optimization adherence differed substantially between trial populations. IVUS-CHIP reported imaging-defined optimization in only 53% of cases, largely due to the technical resistance of heavily calcified lesions, which limited what the imaging findings could realistically achieve, even when acted upon.^19^ Similarly, in the OPTIMAL trial, IVUS data influenced procedural changes in only 29.3% of cases in OPTIMAL, suggesting that experienced operators had already internalized the optimization principles that imaging is designed to enforce.^18^ Importantly, fulfilling prespecified optimization criteria should be distinguished from any operator response to abnormal post-stent IVI findings. In complex lesions, particularly heavily calcified lesions, long or small-vessel lesions, final optimization criteria may be difficult to achieve despite reasonable attempts at correction.^45^ The low rates of procedural change after IVI may indicate limited translation of imaging findings into action. Trial-level reporting of post-stent optimization attempts was inconsistent, preventing formal analysis of this issue. Beyond operator experience and optimization adherence, differences in reference vessel diameter, lesion morphology, and case mix between Asian and Western trial populations may also independently contribute to the geographic interaction and cannot be formally disentangled at the trial level. In our subgroup analysis, we observed that the clinical benefit of IVI was most evident and mainly driven by achieving imaging-defined stent optimization. In this meta-analysis, the choice of imaging modality did not significantly modify the treatment effect. However, modality equivalence in clinical outcomes does not imply that IVUS and OCT are interchangeable across all lesion subtypes. IVUS has advantages in ostial disease, left main PCI, severe calcification, renal dysfunction, and CTO PCI.^10^^,^^44^ OCT provides higher spatial resolution and may be particularly useful for identifying edge dissection, tissue protrusion, malapposition, and detailed mechanisms of stent failure.^10^^,^^44^ Consistent with this is the OCTOBER substudy, done on patients with true unprotected left main bifurcation disease, which found that OCT-guided PCI did not significantly reduce MACE compared with angiography. This is possibly attributable to the technical limitations of OCT at the left main ostium, which include the shortened pullback length and guide catheter shadowing.^46^ A recent side-by-side comparison of DKCRUSH VIII and OCTOBER further highlighted these modality-specific findings in bifurcation PCI.^10^^,^^44^^,^^47^ Operators could consider the modality that is available, familiar, and appropriate for the lesion, while recognizing that the key determinant of benefit is not the imaging catheter itself but the optimization process that follows.^8^^,^^10^^,^^44^ Lesion-type subgroup analyses suggested that the benefit was most seen in left main disease, moderate-to-severe calcification, long lesions, multivessel disease, and ACS presentations. These findings should be considered exploratory since they were based on aggregate subgroup data and were not designed to establish lesion-specific treatment effects.

The present analysis significantly expands and updates prior meta-analyses by incorporating large contemporary trials that materially changed the interpretation of IVI-guided PCI in complex coronary lesions. Stone et al^9^ and Giacoppo et al^15^ reported significant MACE reductions with IVI guidance across broad and unselected PCI populations, but both analyses preceded the publication of IVUS-CHIP, OPTIMAL, and DKCRUSH VIII. Buccheri et al^48^ published a network meta-analysis of 31 studies in 2017 that was among the first to formally compare IVUS, OCT, and angiography across imaging strategies, but it predated the entirety of the contemporary complex-lesion trial generation and did not focus specifically on anatomically complex substrates. Carvalho et al^10^ conducted the most recent lesion-specific network meta-analysis and also did not include these 3 trials. The principal contribution of the present analysis is therefore not a reversal of prior conclusions but a more precise characterization of where IVI benefit is most robust and where it is context-dependent. Notably, extended 5-year follow-up from RENOVATE-COMPLEX-PCI further supports the durability of IVI benefit in complex lesions.^16^ On the other hand, whether specific imaging modalities demonstrate varying levels of benefit within individual lesion subtypes, such as OCT in CTO lesions or IVUS in bifurcation lesions, cannot be determined from trial-level aggregate data and would require pooling of IPD.

### Study limitations

Several limitations should be acknowledged. Publication bias was detected across several outcomes, and trim-and-fill correction attenuated multiple estimates, including MACE, cardiac death, MI, and TLR, to nonsignificance. Leave-one-out analyses showed that some outcomes were sensitive to individual trials, and outcome definitions for MACE and TVF differed across studies, limiting direct comparability. Individual trials diverged on their definitions of severe lesions and also in the optimization criteria. The potential modifying effect of pre-PCI IVI use could not be evaluated because the proportion of pre-PCI imaging was not consistently reported across trials. Similarly, attempted optimization after abnormal post-stent IVI findings could not be assessed because this information was inconsistently reported. This displays a gap that prevents the explanation of this observed geographic interaction and reflects an important deficiency in current trial reporting standards. Moreover, because quantitative coronary angiography was mandated in the angiography arm of only one trial, the impact of systematic quantitative coronary angiography–guided angiographic optimization could not be assessed. Finally, all analyses were based on trial-level aggregate data, limiting the ability to define patient-level or procedural effect modifiers such as lesion complexity, vessel size, operator experience, renal function, and optimization adherence.

## Conclusions

Among patients undergoing PCI for complex coronary lesions, IVI guidance reduces the risk of ischemic events, including stent thrombosis with high consistency, and is associated with lower risks of MACE, cardiac death, MI, and TLR. The benefit might be driven by post-PCI stent optimization. Notably, the clinical benefit was predominantly observed in Asian trial populations, with a significant geographic interaction that warrants further prospective evaluation in Western practice settings. These findings support the adoption of IVI to guide complex PCI.

Perspectives **COMPETENCY IN MEDICAL KNOWLEDGE:** Intravascular imaging guidance reduces ischemic events in complex PCI, particularly stent thrombosis, when imaging-defined stent optimization is achieved.

**COMPETENCY IN PATIENT CARE AND PROCEDURAL SKILLS:** IVUS and OCT should be selected according to lesion characteristics, vessel anatomy, renal function, and operator expertise, with imaging findings translated into effective post-PCI optimization.

**TRANSLATIONAL OUTLOOK:** Future studies should clarify geographic differences in benefit, standardize optimization criteria, and evaluate implementation of imaging-guided complex PCI in Western practice settings.

## Funding support and author disclosures

Dr Brilakis has received consulting/speaker honoraria from Abbott Vascular, American Heart Association (associate editor Circulation), Boston Scientific, Cardiovascular Innovations Foundation (Board of Directors), Cordis, Elsevier, GE HealthCare, Heartflow, IMDS, Medtronic, Recor Medical, SHockwave, SIS Medical, Teleflex, and Terumo; has received research support from AngioWave, Boston Scientific, and GE Healthcare; is the owner of Hippocrates LLC and Systole LLC; and is a shareholder of Cleerly Health, LifeLens Technologies Inc, MHI Ventures, Stallion Medical, and TrueVue Inc. All other authors have reported that they have no relationships relevant to the contents of this paper to disclose.
