# Supplementary material for: Intravascular Imaging-Guided Drug-Eluting Stenting for Complex Coronary Lesions: A Meta-Analysis of Randomized Controlled Trials
Source: JACC Adv. 2026 Oct 15;5(11):103340. doi: 10.1016/j.jacadv.2026.103340 (PMC13694947; doi:10.1016/j.jacadv.2026.103340)
Supplement: Supplementary file 1 [file mmc1.docx]

# **SUPPLEMENTAL MATERIAL**

**Intravascular Imaging-Guided Drug-Eluting Stenting for Complex Coronary Lesions: A Frequentist and Bayesian Meta-Analysis of Randomized Controlled Trials**

Mohammed A. Elbahloul, MD, Bahaa El Deen Wehbeh, MD, Ahmed Samy Badran, MD, Hadeel Aboueisha, MD, Atef Akoum, MD, Ayman Elbadawi, MD, Emmanouil S. Brilakis, MD, Fernando Alfonso, MD, PhD, Islam Y. Elgendy, MD

**Contents**

[**SUPPLEMENTAL MATERIAL**	1](#_Toc238859719)

[Figure S1: Risk of bias assessment according to RoB2.	3](#_Toc238859720)

[Figure S2: Funnel plot for MACE	4](#_Toc238859721)

[Figure S3: Trim and Fill for MACE	5](#_Toc238859722)

[Figure S4: Funnel plot for cardiac death	6](#_Toc238859723)

[Figure S5: Trim and Fill for cardiac death	7](#_Toc238859724)

[Figure S6: Subgroup analysis for MACE based on imaging modality	8](#_Toc238859725)

[Figure S7: Subgroup analysis for MACE based on geographic region	9](#_Toc238859726)

[Figure S8: Sensitivity analysis for MACE excluding studies involving first-generation DES	10](#_Toc238859727)

[Figure S9: Subgroup analysis for cardiac death based on imaging modality	11](#_Toc238859728)

[Figure S10: Subgroup analysis for cardiac death based on geographic region	12](#_Toc238859729)

[Figure S11: Sensitivity analysis for cardiac death excluding studies involving first-generation DES	13](#_Toc238859730)

[Figure S12. Forest plot for all-cause mortality	14](#_Toc238859731)

[Figure S13. Forest plot for myocardial infarction (MI)	15](#_Toc238859732)

[Figure S14. Forest plot for stent thrombosis	15](#_Toc238859733)

[Figure S15. Forest plot for ischemia or clinically driven target lesion revascularization (TLR)	16](#_Toc238859734)

[Figure S16. Forest plot for ischemia or clinically driven target vessel revascularization (TVR)	17](#_Toc238859735)

[Figure S17. Forest plot for target vessel failure (TVF)	18](#_Toc238859736)

[Figure S18. Probability density function illustrating the distribution of treatment effects for major adverse cardiovascular events (MACE).	19](#_Toc238859737)

[Figure S19. Probability density function illustrating the distribution of treatment effects for cardiac death	20](#_Toc238859738)

[Figure S20: Forest plot for stent optimization comparing optimal with suboptimal PCI.	21](#_Toc238859739)

[Figure S21: Leave-one-out for MACE	22](#_Toc238859740)

[Figure S22: Leave-one-out for cardiac death	23](#_Toc238859741)

[Figure S23: Leave-one-out for all-cause mortality	24](#_Toc238859742)

[Figure S24: Leave-one-out for MI	25](#_Toc238859743)

[Figure S25: Leave-one-out for ischemia or clinically driven TVR	26](#_Toc238859744)

[Figure S26: Leave-one-out for ischemia or clinically driven TLR	27](#_Toc238859745)

[Figure S27: Leave-one-out for stent thrombosis	28](#_Toc238859746)

[Figure S28: Leave-one-out for TVF	29](#_Toc238859747)

[Table S1: Search strategy for each database:	27](#_Toc238859748)

[Table S2: Outcomes Definition in each study	30](#_Toc238859749)

[Table S3: Baseline quantitative coronary angiographic data of included studies	33](#_Toc238859750)

[Table S4: The certainty of evidence for each outcome using the Grading of Recommendations Assessment, Development, and Evaluation (GRADE)	34](#_Toc238859751)

[Table S5: Assessment of Publication Bias Using Egger’s Test and Trim-and-Fill Analysis	35](#_Toc238859752)

[Table S6. Bayesian Posterior Probabilities of Risk Reduction for each outcome	36](#_Toc238859753)

[Table S7. Subgroup Analyses for the Composite Outcome of Major Adverse Cardiovascular Events (MACE) or Target Vessel Failure (TVF)	37](#_Toc238859754)

## Figure S1: Risk of bias assessment according to RoB2.

## Figure S2: Funnel plot for MACE

## Figure S3: Trim and Fill for MACE

## Figure S4: Funnel plot for cardiac death

## Figure S5: Trim and Fill for cardiac death

## Figure S6: Subgroup analysis for MACE based on imaging modality

## Figure S7: Subgroup analysis for MACE based on geographic region

## Figure S8: Sensitivity analysis for MACE excluding studies involving first-generation DES

## Figure S9: Subgroup analysis for cardiac death based on imaging modality

## Figure S10: Subgroup analysis for cardiac death based on geographic region

## Figure S11: Sensitivity analysis for cardiac death excluding studies involving first-generation DES

## Figure S12. Forest plot for all-cause mortality

## Figure S13. Forest plot for myocardial infarction (MI)

## Figure S14. Forest plot for stent thrombosis

## Figure S15. Forest plot for ischemia or clinically driven target lesion revascularization (TLR)

## Figure S16. Forest plot for ischemia or clinically driven target vessel revascularization (TVR)

## Figure S17. Forest plot for target vessel failure (TVF)

## Figure S18. Probability density function illustrating the distribution of treatment effects for major adverse cardiovascular events (MACE).

## Figure S19. Probability density function illustrating the distribution of treatment effects for cardiac death

## Figure S20: Forest plot for stent optimization comparing optimal with suboptimal PCI.

## Figure S21: Leave-one-out for MACE

## Figure S22: Leave-one-out for cardiac death

## Figure S23: Leave-one-out for all-cause mortality

## Figure S24: Leave-one-out for MI

## Figure S25: Leave-one-out for ischemia or clinically driven TVR

## Figure S26: Leave-one-out for ischemia or clinically driven TLR

## Figure S27: Leave-one-out for stent thrombosis

## Figure S28: Leave-one-out for TVF

## Table S1: Search strategy for each database:

(PCI OR Percutaneous Coronary Intervention) AND (IVUS OR OCT OR IVI OR Intravascular Ultrasound OR Optical Coherence Tomography OR Intravascular imaging OR intracoronary imaging) AND (Calcified Lesion OR Complex lesions OR Complex coronary lesion OR bifurcation OR long coronary lesion OR CTOs OR ULMCA OR left main coronary artery OR left main)

| Database | Search strategy | Filed | Results |
| --- | --- | --- | --- |
| PubMed | ("PCI"[All Fields] OR ("percutaneous coronary intervention"[MeSH Terms] OR ("percutaneous"[All Fields] AND "coronary"[All Fields] AND "intervention"[All Fields]) OR "percutaneous coronary intervention"[All Fields])) AND ("IVUS"[All Fields] OR "OCT"[All Fields] OR "IVI"[All Fields] OR ("Intravascular"[All Fields] AND ("diagnostic imaging"[MeSH Subheading] OR ("diagnostic"[All Fields] AND "imaging"[All Fields]) OR "diagnostic imaging"[All Fields] OR "ultrasound"[All Fields] OR "ultrasonography"[MeSH Terms] OR "ultrasonography"[All Fields] OR "ultrasonics"[MeSH Terms] OR "ultrasonics"[All Fields] OR "ultrasounds"[All Fields] OR "ultrasound s"[All Fields])) OR ("tomography, optical coherence"[MeSH Terms] OR ("tomography"[All Fields] AND "optical"[All Fields] AND "coherence"[All Fields]) OR "optical coherence tomography"[All Fields] OR ("optical"[All Fields] AND "coherence"[All Fields] AND "tomography"[All Fields])) OR ("Intravascular"[All Fields] AND ("image"[All Fields] OR "image s"[All Fields] OR "imaged"[All Fields] OR "imager"[All Fields] OR "imager s"[All Fields] OR "imagers"[All Fields] OR "images"[All Fields] OR "imaging"[All Fields] OR "imaging s"[All Fields] OR "imagings"[All Fields])) OR ("intracoronary"[All Fields] AND ("image"[All Fields] OR "image s"[All Fields] OR "imaged"[All Fields] OR "imager"[All Fields] OR "imager s"[All Fields] OR "imagers"[All Fields] OR "images"[All Fields] OR "imaging"[All Fields] OR "imaging s"[All Fields] OR "imagings"[All Fields]))) AND ((("calcifiability"[All Fields] OR "calcifiable"[All Fields] OR "calcified"[All Fields] OR "calcifier"[All Fields] OR "calcifiers"[All Fields] OR "calcifies"[All Fields] OR "calcify"[All Fields] OR "calcifying"[All Fields]) AND ("lesion"[All Fields] OR "lesion s"[All Fields] OR "lesional"[All Fields] OR "lesions"[All Fields])) OR (("complex"[All Fields] OR "complex s"[All Fields] OR "complexant"[All Fields] OR "complexants"[All Fields] OR "complexated"[All Fields] OR "complexation"[All Fields] OR "complexations"[All Fields] OR "complexe"[All Fields] OR "complexed"[All Fields] OR "complexes"[All Fields] OR "complexing"[All Fields] OR "complexities"[All Fields] OR "complexity"[All Fields] OR "complexs"[All Fields]) AND ("lesion"[All Fields] OR "lesion s"[All Fields] OR "lesional"[All Fields] OR "lesions"[All Fields])) OR (("complex"[All Fields] OR "complex s"[All Fields] OR "complexant"[All Fields] OR "complexants"[All Fields] OR "complexated"[All Fields] OR "complexation"[All Fields] OR "complexations"[All Fields] OR "complexe"[All Fields] OR "complexed"[All Fields] OR "complexes"[All Fields] OR "complexing"[All Fields] OR "complexities"[All Fields] OR "complexity"[All Fields] OR "complexs"[All Fields]) AND ("coronaries"[All Fields] OR "heart"[MeSH Terms] OR "heart"[All Fields] OR "coronary"[All Fields]) AND ("lesion"[All Fields] OR "lesion s"[All Fields] OR "lesional"[All Fields] OR "lesions"[All Fields])) OR ("bifurcate"[All Fields] OR "bifurcated"[All Fields] OR "bifurcates"[All Fields] OR "bifurcating"[All Fields] OR "bifurcation"[All Fields] OR "bifurcational"[All Fields] OR "bifurcations"[All Fields]) OR ("long"[All Fields] AND ("coronaries"[All Fields] OR "heart"[MeSH Terms] OR "heart"[All Fields] OR "coronary"[All Fields]) AND ("lesion"[All Fields] OR "lesion s"[All Fields] OR "lesional"[All Fields] OR "lesions"[All Fields])) OR "CTOs"[All Fields] OR "ULMCA"[All Fields] OR ("left"[All Fields] AND "main"[All Fields] AND ("coronary vessels"[MeSH Terms] OR ("coronary"[All Fields] AND "vessels"[All Fields]) OR "coronary vessels"[All Fields] OR ("coronary"[All Fields] AND "artery"[All Fields]) OR "coronary artery"[All Fields])) OR ("left"[All Fields] AND "main"[All Fields])) | All Fields | 3760 |
| Scopus | (PCI OR “Percutaneous Coronary Intervention”) AND (IVUS OR OCT OR IVI OR “Intravascular Ultrasound” OR “Optical Coherence Tomography” OR “Intravascular imaging” OR “intracoronary imaging”) AND (“Calcified Lesion” OR “Complex lesions” OR “Complex coronary lesion” OR bifurcation OR “long coronary lesion” OR CTOs OR ULMCA OR “left main coronary artery” OR “left main”) | Article title, Abstract, keywords | 2354 |
| WOS | (PCI OR Percutaneous Coronary Intervention) AND (IVUS OR OCT OR IVI OR Intravascular Ultrasound OR Optical Coherence Tomography OR Intravascular imaging OR intracoronary imaging) AND (Calcified Lesion OR Complex lesions OR Complex coronary lesion OR bifurcation OR long coronary lesion OR CTOs OR ULMCA OR left main coronary artery OR left main) | Topic | 2341 |
| Cochran | (PCI OR Percutaneous Coronary Intervention) AND (IVUS OR OCT OR IVI OR Intravascular Ultrasound OR Optical Coherence Tomography OR Intravascular imaging OR intracoronary imaging) AND (Calcified Lesion OR Complex lesions OR Complex coronary lesion OR bifurcation OR long coronary lesion OR CTOs OR ULMCA OR left main coronary artery OR left main) | Trials | 466 |

## Table S2: Outcomes Definition in each study

| **Study** | **MACE** | **TVF** | **Stent optimization** |
| --- | --- | --- | --- |
| Testa et al. 2026, OPTIMAL | Patient-oriented composite of any stroke, any MI, any revascularization, or death from any cause | device-oriented composite of cardiovascular death, target-vessel MI, or TVR | NA |
| Gao et al. 2026, DKCRUSH VIII | NA | Cardiac death, target vessel MI, or clinically driven TVR | For non–left main lesions, optimal criteria consisted of  the following: 1) the minimum lumen area (MLA) in  the stented segment >5.0 mm2 (side branch or distal  main vessel) and >6.0 mm2 for proximal main vessel  or >90% of the MLA at the distal reference segments;  2) plaque burden 5 mm proximal or distal to the stent  edge was <55%, and 3) no dissection involving the  media >3 mm in length. The following were MLA  criteria for left main lesions: an absolute MLA of  >10 mm2 for the left main, >7 mm2 for the ostial/  proximal left anterior descending artery, or >6 mm2  for the ostial/proximal left circumflex artery (if a 2-stent approach was used for distal left main bifurcation lesions) |
| Diletti et al. 2026, IVUS-CHIP | NA | Composite of cardiac death, target-vessel MI, or clinically indicated TVR | The following criteria were used to define optimal results:  1. Final Minimal stent area (MSA) > 5 mm2 or MSA > 90% of distal reference lumen  2. Plaque burden <50% within 5 mm from the proximal or distal stent edge.  3. No edge dissection involving the media and > 3mm in length |
| Ali et al. 2023, ILUMIEN IV | NA | Cardiac death, Target-vessel MI, Ischemia-driven TVR | OCT-guided stent optimization was performed according to a systematic protocol focused on achieving a maximized Minimum Stent Area (MSA). The criteria included precise vessel sizing (based on proximal and distal reference segments), ensuring adequate stent expansion and apposition to the vessel wall, and the systematic identification and management of procedural complications, such as significant edge dissections and residual atherosclerotic disease at the stent margins |
| Bendary et al. 2024 | MI, TVR, and cardiac death. | NA | NA |
| Chieffo et al. 2013, AVIO | Composite of any MI, cardiac death, and TVR | NA | Stent optimization was defined by a specific IVUS-guided strategy based on the Optimal Balloon Size (OBS). The OBS was determined by averaging the  media-to-media diameters of the distal and proximal stent segments, as well as at the sites of maximal narrowing within the stent. The value was rounded to the lower 0.00 or 0.50 mm. For values 3.5 mm or above, the operator could downsize the balloon diameter as per clinical judgment. Any segment inside the stent with a cross-sectional area (CSA) less than the target criteria for the OBS |
| Gao et al. 2021, Zhang 2018, ULTIMATE | NA | Cardiac death, target vessel MI, and clinically driven TVR | IVUS-defined optimal stent deployment is achieved when there is adequate stent expansion, defined as a minimum lumen area (MLA) > 5.0 mm² or ≥ 90% of the distal reference MLA, along with limited plaque burden at the stent edges (plaque burden < 50% within 5 mm proximal and distal to the stent), and absence of significant edge dissection, specifically no dissection involving the media with a length > 3 mm. |
| Holm et al. 2023, OCTOBER | Cardiac Death, target-lesion MI, or ischemia-driven TLR. | NA | Optimal stent optimization was defined by achieving complete lesion coverage and adequate expansion without complications: the 5-mm stent edge zones had ≤30% diameter stenosis, no major lipid plaque or plaque rupture, and no angiographically visible edge dissection; stent expansion was considered optimal with <10% residual diameter stenosis and <50% stenosis at the side-branch ostium when only the main branch was stented; additionally, there was no stent malapposition (full contact with the vessel wall), and no unintended stent crushing or distortion beyond what was required by the bifurcation technique. |
| Hong et al. 2015, Hong et al. 2020, IVUS-XPL | Cardiac death, target lesion-related MI, or ischemia-driven TLR | NA | IVUS criteria for stent optimization after PCI was defined as a minimal lumen cross-sectional area greater than the lumen cross-sectional area at the distal reference segments. |
| Jakabcin et al. 2010 | Death, MI, and TLR | NA | NA |
| Kim et al. 2013, RESET | Cardiovascular death, MI, TVR, and stent thrombosis. | NA | NA |
| Kim et al. 2015, CTO-IVUS | Cardiac death, MI, and TVR | NA | NA |
| Lee et al. 2023, Lee et al. 2026, RENOVATE-COMPLEX-PCI | NA | Composite of death from cardiac causes, target-vessel–related MI, or clinically driven TVR | NA |
| Li et al. 2024, IVUS-ACS | NA | Composite of cardiac death, target vessel MI, or clinically driven TVR | Optimal stent optimization was defined by IVUS-based criteria requiring all conditions to be met: for non-left main lesions, a minimal stent area >5.0 mm² or ≥90% of the distal reference lumen area, plaque burden <55% within 5 mm of the stent edges, and no medial dissection >3 mm; for left main lesions, target minimal stent areas were >10 mm² in the left main segment, >7 mm² in the ostial/proximal left anterior descending artery, and >6 mm² in the ostial/proximal left circumflex artery (if treated), with all criteria required to be simultaneously satisfied to define optimal stent implantation. |
| Liu et al. 2019 | Composite of cardiac death, MI, and TVR | NA | NA |
| Tan et al. 2015 | Death, non-fatal MI, or TLR | NA | NA |
| Tian et al. 2015, AIR-CTO | All-cause death, MI, TLR, or TVR | NA | IVUS-defined success criteria:  good apposition, stent minimal stent area (MSA) >80% of reference  vessel area, symmetry index >70%, and no >Type B dissection. |
| Hong et al. 2024, OCCUPI | Composite of cardiac death, MI, stent thrombosis, or ischemia-driven TVR | NA | NA |

**NA**: Not Available, **MI:** Myocardial Infarction, **TLR:** target lesion revascularization, **TVR:** target vessel revascularization, TVF: target vessel failure

## Table S3: Baseline quantitative coronary angiographic data of included studies

| Study | Arms | Coronary artery lesion | | | | Reference vessel diameter (mm) [mean ± SD] | Min luminal diameter (mm)  [mean ± SD] | Diameter stenosis % [mean ± SD] | Lesion length (mm) [mean ± SD] |
| --- | --- | --- | --- | --- | --- | --- | --- | --- | --- |
|  |  | LAD, N (%) | LCX, N (%) | RCA, N (%) | Left main, N (%) |  |  |  |  |
| Testa et al. 2026, OPTIMAL | IVUS-Guided | 318/394 (80.7) | 147/394 (37.3) | 55 (14.0) | 384 (97.5) | NA | NA | NA | NA |
|  | Angiography-Guided | 303 (75.2) | 133/403 (33.0) | 56 (13.9) | 395 (98.0) | NA | NA | NA | NA |
| Gao et al. 2026, DKCRUSH VIII | IVUS-Guided | 147 (53.1) | 5 (1.8) | N.A | 124 (44.8) | 3.32 (0.47) | 1.7 (0.81) | 48.27 (24.01) | 11.13 (6.04) |
|  | Angiography-Guided | 146 (52.5) | 8 (2.9) | N.A | 122 (43.9) | 3.34 (0.44) | 1.72 (0.73) | 47.73 (22.3) | 11.53 (6.49) |
| Diletti et al. 2026, IVUS-CHIP | IVUS-Guided | 640 (64.1) | 257 (25.7) | 376 (37.6) | 198 (19.8) | NA | NA | NA | NA |
|  | Angiography-Guided | 648 (64.7) | 280 (27.9) | 356 (35.5) | 177 (17.7) | NA | NA | NA | NA |
| Ali et al. 2023, ILUMIEN IV | OCT-Guided | 704 (53.3) | 251 (19.0) | 365 (27.7) | N.A | 2.93 (0.43) | 0.88 (0.43) | 69.8 (13.9) | 32.9 (15.9) |
|  | Angiography-Guided | 706 (50.9) | 286 (20.6) | 395 (28.5) | N.A | 2.9 (0.42) | 0.88 (0.42) | 69.6 (13.8) | 29.9 (16.1) |
| Bendary et al. 2024 | IVUS-Guided | 86 (95.6) | 47 (52.2) | 15 (16.7) | 90 (100) | N.A | N.A | N.A | N.A |
|  | Angiography-Guided | 90 (98.9) | 55 (60.4) | 17 (18.7) | 91 (100) | N.A | N.A | N.A | N.A |
|  | Angiography-Guided | 70 (63) | 14 (13) | 19 (17) | 8 (7) | 3.25 (0.37) | N.A | 80 (15.04) | 30 (15) |
| Chieffo et al. 2013, AVIO | IVUS-Guided | 97 (53.3) | N.A | N.A | N.A | 2.67 (0.46) | 0.76 (0.46) | 71.6 (15.8) | 27.4 (15.9) |
|  | Angiography-Guided | 87 (48.6) | N.A | N.A | N.A | 2.62 (0.41) | 0.65 (0.45) | 75.5 (16.1) | 25.5 (15) |
| Gao et al. 2021, Zhang 2018, ULTIMATE | IVUS-Guided | 457 (47.5) | 166 (17.3) | 244 (25.4) | 95 (9.9) | N.A | N.A | N.A | 35.1 (21.7) |
|  | Angiography-Guided | 474 (46.7) | 171 (16.8) | 284 (28) | 87 (8.6) | N.A | N.A | N.A | 34.05 (20.7) |
| Holm et al. 2023, OCTOBER | OCT-Guided | 425 (70.8) | 55 (9.2) | 9 (1.5) | 111 (18.5) | 3.3 (0.6) | N.A | 45.2 (20.4) | 20.5 (11.4) |
|  | Angiography-Guided | 422 (70.2) | 56 (9.3) | 7 (1.2) | 116 (19.3) | 3.3 (0.6) | N.A | 41.9 (21.4) | 19.6 (10.7) |
| Hong et al. 2015, Hong et al. 2020, IVUS-XPL | IVUS-Guided | 530 (62.1) | 155 (18.2) | 169 (19.7) | N.A | 2.89 (0.45) | 0.98 (0.2) | 71.1 (14.3) | 34.7 (10.8) |
|  | Angiography-Guided | 535 (63.0) | 152 (17.9) | 162 (19.1) | N.A | 2.85 (0.45) | 0.95 (0.2) | 71.4 (14.4) | 35.2 (10.5) |
| Jakabcin et al. 2010 | Angiography-Guided | 57 (54) | 16 (15) | 25 (24) | 4 (4) | 2.95 (0.34) | 0.97 (0.4) | 79.2 (9.3) | 17.6 (6.7) |
|  | IVUS-Guided | 59 (56) | 12 (11) | 30 (29) | 3 (3) | 3.17 (0.43) | 1.1 (0.4) | 82.3 (7.6) | 18.1 (7.3) |
|  | Angiography-Guided | 32 (33) | 12 (13) | 52 (54) | N.A | N.A | 2.75 (0.5) | N.A | N.A |
| Kim et al. 2013, RESET | IVUS-Guided | 67 (62.1) | 41 (15.2) | 61 (22.7) | NA | 2.85 (0.43) | 0.97 (0.4) | N.A | 31.87 (14.6) |
|  | Angiography-Guided | 185 (67.5) | 35 (12.8) | 54 (19.7) | N.A | 2.84 (0.44) | 0.95 (0.4) | N.A | 31.9 (12.5) |
| Kim et al. 2015, CTO-IVUS | IVUS-Guided | 84 (41.8) | 29 (14.4) | 88 (43.8) | N.A | 2.69 (0.44) | 2.64 (0.35) | 9 (9.8) | 36.3 (17.1) |
|  | Angiography-Guided | 94 (46.8) | 32 (15.9) | 75 (37.3) | N.A | 2.64 (0.55) | 2.56 (0.41) | 10.2 (10.9) | 35.5 (17) |
| Lee et al. 2023, Lee et al. 2026, RENOVATE-COMPLEX-PCI | IVI-Guided | 701 (43.2) | 313 (19.3) | 445 (27.4) | 164 (10.1) | 2.7 (0.5) | 0.44 (0.37) | 85.4 (11.5) | 28.4 (15.9) |
|  | Angiography-Guided | 376 (46.1) | 151 (18.5) | 215 (26.4) | 73 (9.0) | 2.7 (0.4) | 0.44 (0.36) | 85.2 (11.7) | 26.8 (14.8) |
| Li et al. 2024, IVUS-ACS | IVUS-Guided | 1004 (57.3) | 245 (14.0) | 424 (24.2) | 80 (4.6) | 2.98 (0.56) | 1.03 (0.56) | 61.13 (12.54) | 27.77 (14.9) |
|  | Angiography-Guided | 976 (55.7) | 261 (14.9) | 446 (25.5) | 69 (3.9) | 3 (0.59) | 1.04 (0.56) | 60.5 (12.25) | 27.54 (14.47) |
| Liu et al. 2019 | IVUS-Guided | 93 (55.7) | 74 (44.3) | N.A | 167 (100) | N.A | N.A | N.A | N.A |
|  | ICA-Guided | 89 (52.7) | 84 (49.7) | N.A | 169 (100) | N.A | N.A | N.A | N.A |
| Tan et al. 2015 | Angiography-Guided | N.A | N.A | N.A | 61 (100) | N.A | 1.92 (0.2) | N.A | N.A |
|  | IVUS-Guided | N.A | N.A | N.A | 62 (100) | N.A | 1.9 (0.2) | N.A | N.A |
| Tian et al. 2015, AIR-CTO | Angiography-Guided | 42 (36.5) | 17 (14.8) | 53 (46.1) | 3 (2.6) | 2.6 (0.3) | N.A | N.A | 30.6 (18.74) |
|  | IVUS-Guided | 51 (44.3) | 24 (20.9) | 40 (34.8) | N.A | 2.65 (0.4) | N.A | N.A | 29 (17.4) |
| Hong et al. 2024, OCCUPI | OCT-Guided | N.A | N.A | N.A | N.A | 2.8 (0.5) | 0.7 (0.4) | N.A | 35.47 (18) |
|  | Angiography-Guided | N.A | N.A | N.A | N.A | 2.8 ( 0.5) | 0.7 (0.5) | N.A | 34.3 (17.5) |

N.: number, N.A: not available, LAD: Left Anterior Descending Artery, LCX: Left Circumflex Artery, RCA: Right Coronary Artery, IVI: Intravascular imaging, IVUS: Intravascular ultrasound.

## Table S4: The certainty of evidence for each outcome using the Grading of Recommendations Assessment, Development, and Evaluation (GRADE)

| **Certainty assessment** | | | | | | | **Summary of findings** | | | | |
| --- | --- | --- | --- | --- | --- | --- | --- | --- | --- | --- | --- |
| **Participants (studies) Follow-up** | **Risk of bias** | **Inconsistency** | **Indirectness** | **Imprecision** | **Publication bias** | **Overall certainty of evidence** | **Study event rates (%)** | | **Relative effect (95% CI)** | **Anticipated absolute effects** | |
|  |  |  |  |  |  |  | **With angiography** | **With IVI** |  | **Risk with angiography** | **Risk difference with IVI** |
| **MACE** | | | | | | | | | | | |
| 7320 (12 RCTs) | not serious | not serious | not serious | not serious | publication bias strongly suspected^a^ | ⨁⨁⨁◯ Moderate^a^ | 517/3666 (14.1%) | 377/3654 (10.3%) | **RR 0.67** (0.55 to 0.83) | 517/3666 (14.1%) | **47 fewer per 1,000** (from 63 fewer to 24 fewer) |
| **Cardiac death** | | | | | | | | | | | |
| 13608 (14 RCTs) | not serious | not serious | not serious | not serious | publication bias strongly suspected^a^ | ⨁⨁⨁◯ Moderate^a^ | 160/6537 (2.4%) | 143/7071 (2.0%) | **RR 0.72** (0.53 to 0.97) | 160/6537 (2.4%) | **7 fewer per 1,000** (from 12 fewer to 1 fewer) |
| **All-cause mortality** | | | | | | | | | | | |
| 11856 (12 RCTs) | not serious | not serious | not serious | serious^b^ | none | ⨁⨁⨁◯ Moderate^b^ | 258/5660 (4.6%) | 267/6196 (4.3%) | **RR 0.88** (0.75 to 1.04) | 258/5660 (4.6%) | **5 fewer per 1,000** (from 11 fewer to 2 more) |
| **MI** | | | | | | | | | | | |
| 12599 (15 RCTs) | not serious | not serious | not serious | not serious | publication bias strongly suspected^a^ | ⨁⨁⨁◯ Moderate^a^ | 401/6033 (6.6%) | 370/6566 (5.6%) | **RR 0.87** (0.75 to 0.998) | 401/6033 (6.6%) | **9 fewer per 1,000** (from 17 fewer to 0 fewer) |
| **Ischemia or clinically driven TVR** | | | | | | | | | | | |
| 7845 (5 RCTs) | not serious | serious^c^ | not serious | not serious | publication bias strongly suspected^a^ | ⨁⨁◯◯  low^a,c^ | 209/3922 (5.3%) | 178/3923 (4.5%) | **RR 0.69** (0.43 to 1.12) | 209/3922 (5.3%) | **17 fewer per 1,000** (from 30 fewer to 6 more) |
| **Ischemia or clinically driven TLR** | | | | | | | | | | | |
| 6428 (5 RCTs) | not serious | serious^c^ | not serious | not serious | none | ⨁⨁⨁◯ Moderate^c^ | 181/3217 (5.6%) | 135/3211 (4.2%) | **RR 0.72** (0.51 to 1.00) | 181/3217 (5.6%) | **16 fewer per 1,000** (from 28 fewer to 0 fewer) |
| **Stent thrombosis** | | | | | | | | | | | |
| 11988 (13 RCTs) | not serious | not serious | not serious | not serious | none | ⨁⨁⨁⨁ High | 86/5728 (1.5%) | 45/6260 (0.7%) | **RR 0.52** (0.35 to 0.78) | 86/5728 (1.5%) | **7 fewer per 1,000** (from 10 fewer to 3 fewer) |
| **TVF** | | | | | | | | | | | |
| 7485 (5 RCTs) | not serious | serious^c^ | not serious | not serious | none | ⨁⨁⨁◯ Moderate^c^ | 413/3472 (11.9%) | 444/4013 (11.1%) | **RR 0.85** (0.61 to 1.19) | 413/3472 (11.9%) | **18 fewer per 1,000** (from 46 fewer to 23 more) |

CI: Confidence Interval, IVI: Intravascular Imaging, MACE: Major Adverse Cardiovascular Events, MI: Myocardial Infarction, RCT: Randomized Controlled Trial, RR: Risk Ratio, TLR: Target Lesion Revascularization, TVF: Target Vessel Failure, TVR: Target Vessel Revascularization

Explanations

a. Significant Asymmetry

b. Inconsistent direction of effect

c. The 95% confidence interval crosses the null effect

## Table S5: Assessment of Publication Bias Using Egger’s Test and Trim-and-Fill Analysis

| Outcome | No. of included studies | Egger’s P-value | Publication Bias | No. of Imputed Studies | Adjusted RR (95% CI) by Trim-and-Fill Analysis |
| --- | --- | --- | --- | --- | --- |
| MACE | 12 | 0.004 | Yes | 6 | 0.85 (0.67-1.08)* |
| Cardiac death | 14 | 0.01 | Yes | 6 | 0.85 (0.62-1.16)* |
| All-cause mortality | 12 | 0.63 | No | 0 | 0.88 (0.75-1.04) |
| MI | 15 | 0.02 | Yes | 5 | 0.91 (0.79-1.04)* |
| Ischemia or clinically driven TVR | 5 | 0.02 | Yes | 2 | 0.98 (0.54-1.78) |
| Ischemia or clinically driven TLR | 5 | 0.21 | No | 2 | 0.89 (0.60-1.31)* |
| Stent thrombosis | 13 | 0.48 | No | 2 | 0.57 (0.38-0.84) |
| TVF | 5 | 0.10 | No | 1 | 0.97 (0.64-1.46) |

CI: Confidence Interval, MACE: Major Adverse Cardiovascular Events, MI: Myocardial Infarction, RR: Risk Ratio, TLR: Target Lesion Revascularization, TVF: Target Vessel Failure, TVR: Target Vessel Revascularization

*Became no longer significant after being adjusted by the Trim-and-Fill analysis

## Table S6. Bayesian Posterior Probabilities of Risk Reduction for each outcome

| Outcome | Posterior Probabilities for RR > 1 | Posterior Probabilities for RR < 1 | Posterior Probabilities for RR < 0.90 | Posterior Probabilities for RR < 0.80 |
| --- | --- | --- | --- | --- |
| MACE | 0 | 100% | 99.7% | 79.5% |
| Cardiac death | 1.7% | 98.3% | 88.0% | 54.3% |
| All-cause mortality | 7.2% | 92.8% | 58.2% | 12% |
| MI | 1.6% | 98.4% | 72.3% | 13.6% |
| Ischemia, or clinically driven TVR | 6.7% | 93.3% | 67.6% | 23.1% |
| Ischemia or clinically driven TLR | 0.5% | 99.5% | 94.4% | 69.7% |
| Stent thrombosis | 0 | 100% | 99.8% | 99.8% |
| TVF | 14.9% | 85.1% | 27.5% | 0.6% |

MACE: Major Adverse Cardiovascular Events, MI: Myocardial Infarction, RCT: Randomized Controlled Trial, RR: Risk Ratio, TLR: Target Lesion Revascularization, TVF: Target Vessel Failure, TVR: Target Vessel Revascularization

## Table S7. Subgroup Analyses for the Composite Outcome of Major Adverse Cardiovascular Events (MACE) or Target Vessel Failure (TVF)

| Subgroup | No. of included studies | event rates (%) in IVI | event rates (%) in Angio | | RR, 95% CI | 95% PI | Heterogeneity |
| --- | --- | --- | --- | --- | --- | --- | --- |
| LMCAD | 9 | 251/1407 | 288/1313 | | **0.64 (0.44-0.91)** | 0.21-1.91 | I^2^ = 72.4%, P < 0.01 |
| CTOs | 6 | 82/935 | 99/806 | | 0.67 (0.41- 1.08) | 0.17-2.53 | I^2^ = 60.6%, P = 0.03 |
| Bifurcation lesions | 7 | 189/1886 | 236/1819 | | 0.73 (0.51-1.05) | 0.26-2.06 | I^2^ = 68.8%, P < 0.01 |
| Moderate to severe Calcified lesions | 5 | 145/1164 | 159/1044 | | 0.71 (0.50-1.02) | 0.26-1.92 | I^2^ = 61.4%, P = 0.03 |
| Long coronary lesions | 7 | 295/4066 | 350/3690 | | **0.70 (0.53-0.93)** | 0.31-1.58 | I^2^ = 72.2%, P < 0.01 |
| MVD | 6 | 136/1791 | 213/1689 | | **0.58 (0.46-0.73)** | 0.39-0.87 | I^2^ = 4.3%, P = 0.39 |
| ISR | 4 | 83/541 | 86/487 | | 0.86 (0.65-1.14) | 0.55-1.36 | I^2^ = 0%, P = 0.63 |
| Clinical presentation | | | | P _interaction_ = 0.88 | | | |
| ACS | 10 | 393/3897 | 452/3571 | | **0.74 (0.60-0.92)** | 0.38-1.44 | I^2^ = 61.4%, P < 0.01 |
| Non-ACS | 8 | 295/3312 | 334/3135 | | 0.76 (0.58-1.01**)** | 0.33-1.74 | I^2^ = 69.6%, P < 0.01 |
| Sex | | | | P _interaction_ = 0.75 | | | |
| Male | 8 | 500/4773 | 512/4319 | | 0.78 (0.59-1.02) | 0.33-1.86 | I^2^ = 77.9%, P < 0.01 |
| Female | 8 | 121/1343 | 155/1276 | | **0.73 (0.57-0.95)** | 0.44-1.22 | I^2^ = 16.3%, P = 0.30 |
| Age | | | | P _interaction_ = 0.99 | | | |
| < 65 | 6 | 181/2396 | 204/2136 | | **0.76 (0.59-0.99)** | 0.39-1.48 | I^2^ = 45.2%, P = 0.10 |
| ≥ 65 | 6 | 288/3042 | 297/2776 | | 0.80 (0.62-1.04) | 0.40-1.62 | I^2^ = 62.3%, P = 0.02 |
| < 75 | 5 | 236/2325 | 250/2359 | | 0.77 (0.47-1.26) | 0.16-3.75 | I^2^ = 63.1%, P < 0.01 |
| ≥ 75 | 5 | 125/977 | 132/939 | | 0.73 (0.45-1.19) | 0.18-2.97 | I^2^ = 84%, P = 0.03 |

ACS: Acute Coronary Syndrome, CI: Confidence Interval, CTOs: Chronic Total Occlusions, ISR: In-Stent Restenosis, IVI: Intravascular Imaging, LMCAD: Left Main Coronary Artery Disease, MACE: Major Adverse Cardiovascular Events, MVD: Multivessel Disease, PI: Prediction Interval, RR: Risk Ratio, TVF: Target Vessel Failure
